# The *Escherichia coli* Serogroup O1 and O2 Lipopolysaccharides Are Encoded by Multiple O-antigen Gene Clusters

**DOI:** 10.3389/fcimb.2017.00030

**Published:** 2017-02-07

**Authors:** Sabine Delannoy, Lothar Beutin, Patricia Mariani-Kurkdjian, Aubin Fleiss, Stéphane Bonacorsi, Patrick Fach

**Affiliations:** ^1^IdentyPath Platform, Food Safety Laboratory, Anses, Université Paris-EstMaisons-Alfort, France; ^2^National Reference Laboratory for Escherichia coli, Federal Institute for Risk Assessment (BfR)Berlin, Germany; ^3^Department of Biology, Chemistry, Pharmacy, Institute for Biology - Microbiology, Freie Universität BerlinBerlin, Germany; ^4^CNR Associé Escherichia coli, Service de Microbiologie, Hôpital Robert-DebréParis, France; ^5^IAME, UMR 1137, INSERMParis, France; ^6^IAME, UMR 1137, University Paris Diderot, Sorbonne Paris CitéParis, France

**Keywords:** *E. coli*, extraintestinal *E. coli*, ExPEC, Shiga toxin-producing *E. coli*, STEC, serotyping, O-antigen gene cluster, O-AGC

## Abstract

*Escherichia coli* strains belonging to serogroups O1 and O2 are frequently associated with human infections, especially extra-intestinal infections such as bloodstream infections or urinary tract infections. These strains can be associated with a large array of flagellar antigens. Because of their frequency and clinical importance, a reliable detection of *E. coli* O1 and O2 strains and also the frequently associated K1 capsule is important for diagnosis and source attribution of *E. coli* infections in humans and animals. By sequencing the O-antigen clusters of various O1 and O2 strains we showed that the serogroups O1 and O2 are encoded by different sets of O-antigen encoding genes and identified potentially new O-groups. We developed qPCR-assays to detect the various O1 and O2 variants and the K1-encoding gene. These qPCR assays proved to be 100% sensitive and 100% specific and could be valuable tools for the investigations of zoonotic and food-borne infection of humans with O1 and O2 extra-intestinal (ExPEC) or Shiga toxin-producing *E. coli* (STEC) strains.

## Introduction

Strains of the *E. coli* species are currently divided into 183 O-groups (lipopolysaccharide) and 53 H-types (flagellar antigen) (Joensen et al., 2015). For many decades serotyping of O- and H-antigens and in some cases capsular (K-antigens) was the only way to classify *E. coli* which shared similarities in their fermentation reactions, hosts, virulence markers, and their pathogenicity to humans or animals (Orskov and Orskov, 1984, 1992; Edwards and Ewing, 1986). Most of the genes responsible for the biosynthesis of the O-antigen are located in a single cluster, the O-antigen gene cluster or O-AGC (Reeves et al., 1996; Iguchi et al., 2015a; DebRoy et al., 2016). The size and gene content of the O-AGC vary between serogroups, and two genes, *wzx* (O-antigen flippase) and *wzy* (O-antigen polymerase) or *wzm* (O-antigen ABC transporter permease gene) and *wzt* (ABC transporter ATP-binding gene), appear highly specific for each serogroup. These can serve as targets for molecular determination of the serogroup by PCR, qPCR or nucleotide sequencing (Iguchi et al., 2015b; Joensen et al., 2015; Fratamico et al., 2016). The conventional serotype identification, based on the serological agglutination of the O-antigens and flagellar H-antigens, is difficult, time consuming and expensive. In addition, many samples remain untypeable by agglutination. Indeed, cross-reactions, the existence of “O rough” strains that have lost their ability to express the O antigen, and non-motile strains that do not express a flagellar antigen, all hamper the efficiency of the method. Molecular serotyping, targeting genes involved in surface antigen synthesis, appears to be a rapid, specific and cheaper alternative to conventional serotyping.

*Escherichia coli* strains belonging to serogroups O1 and O2 were among the first 20 O-serogroups defined when the serotyping scheme was introduced by Kauffmann in 1944 (Orskov and Orskov, 1984). The prototype strains for the *E. coli* O1 and O2 serogroups were from human extra-intestinal infections (Orskov and Orskov, 1984). *Escherichia coli* O1 and O2 strains were since found to be associated with disease in humans and animals and also heterogeneous for their hosts, phenotypical traits and the disease they cause in humans and animals. Certain *E. coli* O1 and O2 strains such as O1:K1:H7 or O2:K1:H4, O2:K1:H5, O2:K1:H6, and O2:K1:H7 were identified as causative agents of urinary tract infection, septicaemia, and neonatal meningitis in humans and animals (Orskov, 1978; Achtman et al., 1986; Olesen et al., 1995). Both serogroups account for the majority of strains causing bacteraemia and sepsis in human patients (Orskov and Orskov, 1992; Olesen et al., 1995).

*Escherichia coli* O1:K1:H7, O2:K1:H4, O2:K1:H5, and O2:K1:H7 strains belong to a clonal group (sequence type 95) frequently associated with extra-intestinal diseases in humans and poultry (Dziva and Stevens, 2008; Riley, 2014). These extra-intestinal pathogenic *E. coli* (ExPEC) constitute an emerging public health problem (Johnson et al., 2012a), in particular if they carry plasmids encoding resistance to antimicrobial agents. Meanwhile, the avian pathogenic *Escherichia coli* (APEC) strains, associated with colibacillosis, a serious illness in poultry worldwide, are the cause of significant economic losses in poultry farms. Poultry strains share some ExPEC virulence traits with human O1:K1:H7 or O2:K1:H7 isolates and with other ExPEC strains frequently isolated from human patients (Johnson et al., 2006, 2007, 2012b; Dziva and Stevens, 2008; Houdouin et al., 2008; Mora et al., 2009; Peigne et al., 2009; Nandanwar et al., 2014). *Escherichia coli* O1:H6 strains form another group of ExPEC that were isolated from extra-intestinal infections of dogs, cats and horses (Ewers et al., 2014; Guo et al., 2015).

The human gut microbiota constitutes an important reservoir of *E. coli* O1:K1:H1, O1:K1:H7 and O2:K1:H4 and these strains can thus serve as source of extra-intestinal infections (Damborg et al., 2009; Moreno et al., 2009). A possible route of human contamination is through the food supply with an initial colonization of the intestinal tract before onset of an extra-intestinal infection (Manges and Johnson, 2015). Indeed, apart from poultry, many of these ExPEC strains sharing virulence markers with human isolates were isolated from a variety of sources, including animals, food, and the environment (Jakobsen et al., 2010; Singer, 2015). O2 strains such as O2:H6 or O2:H42, are frequently isolated from cattle (Diarra et al., 2009). *Escherichia coli* O2:H7 similar to isolates causing human infections were also isolated from food (Vincent et al., 2010).

Some *Escherichia coli* O1 and O2 strains producing Shiga toxins (STEC) such as O1:H20, O1:H7, O1:H2, and O1:H1 were sporadically isolated from feces of human patients with diarrhea and STEC O2:K1:H7 have caused sporadic cases of HUS in humans (Kobayashi et al., 1993; Beutin et al., 2004; Blanco et al., 2004; Chen et al., 2014). Cattle most likely serves as a reservoir for these strains as STEC O1:H20 and other STEC O1 strains were isolated from bovine meat products and cattle feces (Hussein and Bollinger, 2005; Mekata et al., 2014) while STEC O2:H27 and other STEC O2 have been recovered from feces of cattle, pigs and wild ruminants (Sánchez et al., 2009; Scott et al., 2009; Hutchinson et al., 2011). Interestingly, poultry, pigs and sheep can also harbor enteropathogenic *E. coli* (EPEC) O2 strains such as O2:H40, O2:H49, or O2:H8 (Fröhlicher et al., 2008; Alonso et al., 2016).

By analyzing different types of O1 strains, five different serological factors (O1a–O1e) were described (Moll et al., 1986) and three different polysaccharide structures (O1A, O1B, and O1C) were characterized which differ slightly in their composition (Gupta et al., 1992). The O1A type corresponded to *E. coli* O1:K1:H7 strains from human patients. The closely related O1B and O1C types were associated with other types of *E. coli* O1 strains (Achtman et al., 1983; Moll et al., 1986; Gupta et al., 1992). These differences in the LPS structures suggest that serogroup O1 strains show alterations in their O-antigen encoding genes. Moreover, serogroup O1 was reported to show multiple cross-reactions with other O-serogroups such as O2, O10, O14, O50, O53, O64, O70, O107, O115, O117, O148, O149, and O154 (Orskov and Orskov, 1984; Edwards and Ewing, 1986). The O2 antisera can also give cross-reactions with serogroups O1, O50, O53, O74, and O117 (Edwards and Ewing, 1986).

In this work, we investigated ExPEC, EPEC, and STEC strains of serogroup O1 and O2 for their O-antigen encoding genes to explore the nature of different antigens described for O1 and to explain the cross-reactivity between O1, O2, and other *E. coli* O-groups. As the nucleotide sequences of O-antigen encoding genes O1-O186 are now available (Iguchi et al., 2015a; DebRoy et al., 2016) we developed a qPCR-based detection procedure to investigate O1 and O2 strains of different H-types and from various sources. By sequencing the O-AGC of non-reacting strains, we showed that the serogroups O1 and O2 are encoded by different sets of O-antigen encoding genes and identified potentially new serotypes. The capsular antigen K1 contributes to the survival of ExPEC strains in the blood stream and is frequently associated with strains causing septicaemia and meningitis. We developed an additional qPCR assay for the specific detection of the capsular antigen K1 often associated with the clinically highly important O1 and O2 and other ExPEC strains.

## Materials and methods

### Bacteria

*Escherichia coli* strains used in this study were derived from the collections of the National Reference Laboratory for *E. coli* (NRL *E. coli*) at the Federal Institute for Risk Assessment (BfR) in Berlin, Germany, the associated French National Reference Center for *E. coli* at the pediatric Hospital Robert-Debré in Paris (France), and from the French Agency for Food, Environmental and Occupational Health and Safety (Anses) in Maisons-Alfort, France.

Most of the strains studied here belong to previously described collections (Wullenweber et al., 1993; Beutin et al., 2004, 2007, 2016; Bidet et al., 2007; Bugarel et al., 2010; Martin and Beutin, 2011). *E. coli* strains used to test for the specificity of the qPCR assays included in particular the *E. coli* reference strains belonging to serogroups O1-O186 and H-types H1-H56. *E. coli* strains carrying the capsular antigen K1 (Orskov and Orskov, 1984; Wullenweber et al., 1993; Bettelheim et al., 2003) were used as reference strains for the *neuB* gene specific real-time PCR described in Table 1.

**Table 1:** **Primers and probes for the real-time PCR assays**.

**Target gene**	**Forward primer, reverse primer, and probe sequences (5′–3′)**
*wzy*O1A	CCTTTTGTATTTTCTTTCTGGCTAGTG
	CGAATATTTTATCCGATGGCTTTAG
	[6FAM]- AGTCCCGGTCATTGCTTATCGAACTGC-[BHQ1]
*wzx*O1non-A	AATCATTTGATGTCGGCATGTC
	GATTTTTATACTTACATGGTGGATCGTATC
	[6FAM] -TCAGCTATACGCACTGGGCGTCCC-[BHQ1]
*wzxO2*	GCCAAGTGCAAAGTTTAATCACAAT
	CTTGCCAATTTTCCGCAGTATAT
	[6FAM]- CCTCTGCACCTGTAAGCACTGGCCTT-[BHQ1]
*wzxO2-2 (CB11127)*	TCCGTTATATTTTGATGGCATTGA
	CCCTGTTACATTCCACCCTTCT
	[6FAM]-CAGCGCATTAGTTTTTCCACTTGCCTTG-[BHQ1]
*wzxO2-3 (CB15123)*	CCACCACGGTGCACATTTAC
	GGACAGGTACAAAGCCTAATGAATATT
	[6FAM]-TTTCCCTTTACCTCCATCCCAATTTTCTGC-[BHQ1]
*neuB*K1	TCAATAGAACCTGATGAACTGAAACAT
	TCTGATCATTCTAGCGGGTTTTTATG
	[6FAM] –TTATTCCATAAGGCACCGCCGCAA – [BHQ1]

Two STEC O1 strains, CB13533 (O1:H33) and CB14293 (O1:H20), the *E. coli* O1:H12 strain CB11070, the O1 reference strain A47 (O1:HNT), the O50 reference strain U18-41 and two EPEC O2 strains CB11127 (O2:H49) and CB15123 (O2:H40) were investigated for the gene sequences of their O-antigen locus (Table 2).

**Table 2:** **Properties of the ***E. coli*** strains used for sequencing of the O-AGC**.

**Strain**	**Source**	**Year isolated or reference**	Serotype (as defined by agglutination)^*^	**stx1**	**stx2**	**eae**	**Accession number**
CB11070	Pig feces	2007	O1:H12	−	−	−	KY115223
CB13533	Bovine feces	2011	O1:H33	+	−	−	KY115224
CB14293	veal	2012	O1:H20	+	−	−	KY115225
A47	reference strain, pig feces	Moll et al., 1986	O1:[H21]	−	−	−	KY115226
U18-41	Human urine	1944	O50:H4	−	−	−	KY115227
CB11127	pig	Fröhlicher et al., 2008	O2:H49	−	−	+	KY115228
CB15123	Chicken meat	2013	O2:H40	−	−	+	KY115229

* *Serotypes between brackets were determined with a qPCR-based assay*.

All strains were grown overnight at 37°C in Luria broth, and DNA was extracted according to the manufacturer's instructions using InstaGene matrix (BioRad laboratories, Marnes-La-Coquette, France).

### Serotyping of *E. coli* strains and detection of capsular antigen K1

Serotyping of O (lipopolysaccharide) and H (flagellar) antigens of the strains received at the BfR was performed with O-specific and H-specific rabbit antisera prepared according to standard methods (Orskov and Orskov, 1984). The O1 and O2 serogroups reference strains U5-41 (O1:K1:H7) and U9-41 (O2:K1:H4) (Orskov and Orskov, 1984) were used for preparation of *E. coli* O1 and O2 antisera following previously published protocols (Orskov and Orskov, 1984; Edwards and Ewing, 1986). Two-fold dilutions of O-antiserum in a range 1:50–1:12,800 were used for testing boiled cultures of bacteria for agglutination reactions. Agglutination reactions ± one titer step as obtained with the positive control strains U5-41 and U9-41 (both end point titer 1:12,800) were interpreted as positive for serogroup O1 or O2 respectively following published serotyping methods (Orskov and Orskov, 1984; Edwards and Ewing, 1986). Strains carrying the K1 capsule were detected using K1 specific bacteriophages as previously described (Wullenweber et al., 1993).

Clinical strains isolated at the Robert Debré hospital were either serotyped by agglutination as described above or serogrouped using a multiplex PCR assay (Clermont et al., 2007). The K1 antigen was detected using the Wellcogen™ Bacterial Antigen Rapid Latex Agglutination Test according to the manufacturer's instructions.

### PCR detection and mapping of *E. coli* O-antigen genes

Nucleotide sequence data obtained from the previously published sequence of the lipopolysaccharide O1 and O2 antigens (Li et al., 2010) and capsular K1 antigen (Lu et al., 2011) encoding genes were used for designing TaqMan® qPCR probes for specific detection of *E. coli* O1-, O2-, and K1-antigen encoding strains. As some of the investigated O1 and O2 strains did not react with the wzyO1A and wzxO2 qPCR assays, the nucleotide sequence of the O-antigen encoding genes of four qPCR-negative O1 strains (A47, CB11070, CB13533, and CB14293) and two qPCR-negative O2 strains (CB11127 and CB15123) were determined (see below). A qPCR for the variant *wzx* gene present in O1 strains CB13533 and CB14293 was developed and used for further typing of the collection of *E. coli* O1 strains. In a similar way, two qPCRs for the variant *wzx* genes of CB11127 and CB15123 were developed and used for molecular typing of *E. coli* O2 strains (Table 1). Real-time qPCR probes and primers used in this work were designed with the software Primer Express V3.0 (Applied Biosystems) and are described in Table 1.

Real-time PCR assays were performed with an ABI7500 instrument (Applied Biosystems, Foster City, CA, USA) in 25-μl reaction volumes, a Light Cycler 1536 (Roche Diagnostics, Meylan, France) in 1.5-μl reaction volumes or a BioMark (Fluidigm, South San Fransisco, CA, USA) in nanofluidic format according to the recommendations of the suppliers. Primers and TaqMan probes were used at 300 nM final concentrations. The following two-steps thermal profile was applied to all instruments: enzyme activation at 95°C for 1–10 min (as recommended depending on the enzyme used), followed by 40 cycles of denaturation at 95°C and annealing at 60°C.

### Nucleotide sequencing

Genomic DNA of strains CB11070, CB13533, CB14293, A47, CB11127, CB15123, and of the O50 reference strain U18-41 was extracted from an overnight culture in tryptic soy broth (TSB) medium using the DNeasy blood and tissue kit (Qiagen). The O-antigen cluster of the strains was amplified by long range PCR between the *galF* and *gnd* genes, using the Expand Long Range PCR system (Roche) and either the 1523/1524 primers (A47, CB14293, CB13533, CB11127), 412/482 primers (CB15123), or 412/482b primers (CB11070) as described previously (Coimbra et al., 2000; Guo et al., 2004; Perelle et al., 2005). The amplicons were purified using the Charge Switch kit (Invitrogen) and used to prepare libraries with the Nextera XT kit (Illumina) according to the recommendations of the manufacturers. Paired-end short-read sequencing was performed using an Illumina MiSeq instrument (Illumina), according to the manufacturer's instruction. The raw reads were trimmed (minimum length, 35 bp; quality score, 0.03) and assembled in CLC Genomics Workbench 7.5.1 by *de novo* assembly (minimum contig length, 1000 bp), producing a single contig for each sample. The sequences were annotated using PROKKA (Seemann, 2014) on a Galaxy platform. Unannotated ORFs were further manually curated and analyzed by functional domain analysis using InterPro (Mitchell et al., 2015) and Blastp.

The annotated O-antigen cluster sequences of these strains were deposited in DDBJ/ENA/GenBank under the accession numbers listed in Table 2.

## Results

### Analysis of the O-antigen encoding genes in *E. coli* O1 strains

A qPCR was developed for the *E. coli wzy*O1 gene derived from the sequence of the *E. coli* O1 strain G1632 (U5-41) (Li et al., 2010; GenBank Accession GU299791; Table 1). This PCR was tested on an initial panel of 36 *E. coli* O1 strains and reacted only with 17 of these (Table 3, see below). The O1 antigen encoding genes of four non-reacting strains, A47 (O1:H-/[H21]), CB11070 (O1:H12), CB13533 (O1:H33), and CB14293 (O1:H20), were analyzed by nucleotide sequencing.

**Table 3:** **Investigation of ***E. coli*** O1 strains by qPCR with O1 and K1 specific assays**.

**Serotype^a^**	**Source, origin**	**Reference/year isolated**	**Stx**	**Nos of strains**	***wzy*_O1A_**	***wzx*_O1non−A_**	***neuBK1***
O1:K1:H7	See footnote b	See footnote b	−	11	+	−	+
O1:H31	Sheep feces, Norway	Döpfer et al., 2010	−	1	+	−	−
O1:[H34]	Cattle feces, Germany	This work, 2008	−	1	+	−	−
O1:H42	Human feces, France	This work, 2009	−	1	+	−	−
O1:H6	Human feces, France, Switzerland	Geser et al., 2012	−	2	+	−	−
O1:H10	Cattle feces, Germany	This work, 2011	Stx1	1	+	−	−
O1:H12	Pig feces, Germany	This work, 2007	−	1	−	+	−
O1:H20	See footnote c	See footnote c	Stx1	15	−	+	−
O1:H33	Cattle feces, Germany	This work, 2011	Stx1	1	−	+	−
O1:H19	Pig feces, Germany	Beutin et al., 1993	−	1	−	+	−
O1:NM	A47, O1non-A reference strain, pig feces	Moll et al., 1986; Gupta et al., 1992	−	1	−	+	−

a Serotypes between brackets were determined by fliC gene sequencing.

b Five O1:K1:H7 strains were from extraintestinal infections of humans (Achtman et al., 1983) including the serogroup reference strains U5-41 (Orskov and Orskov, 1984), A220 and A25 (Achtman et al., 1983; Moll et al., 1986). Four O1:K1:H7 strains were from diseased poultry and two from feces of healthy infants (Bettelheim et al., 2003).

c *Thirteen of the 15 O1:H20 strains were positive for stx_1_. One of these was also positive for stx2. An additional strain was positive only for stx2. Eight O1:H20 strains were from bovine meat products and isolated in Germany between 2010 and 2012. One strain was from lamb meat product and isolated in Germany in 2008.Two O1:H20 strains were feces of cattle (Germany, 2011) and three from stool of humans with diarrhea (Beutin et al., 2004). The only O1:H20 strain that was Stx-negative was isolated from the feces of a healthy dog (Beutin et al., 1993)*.

**Figure 1:**
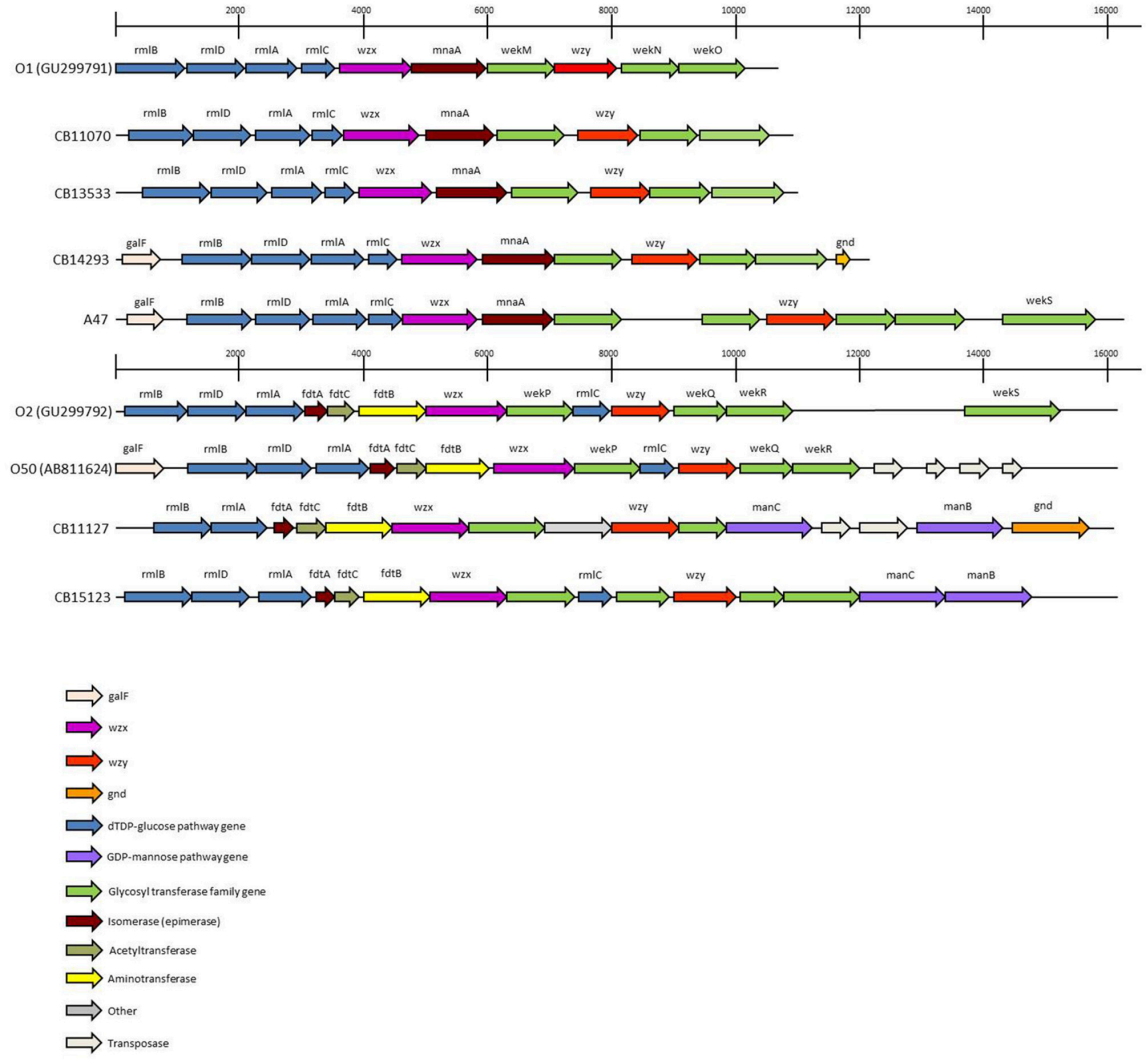
**The O-Antigen gene clusters of O1, O2 and O50 strains and variants thereof**. The accession numbers of sequences obtained from public databases are indicated. The open arrows represent the location and orientation of the putative genes identified.

**Figure 2:**
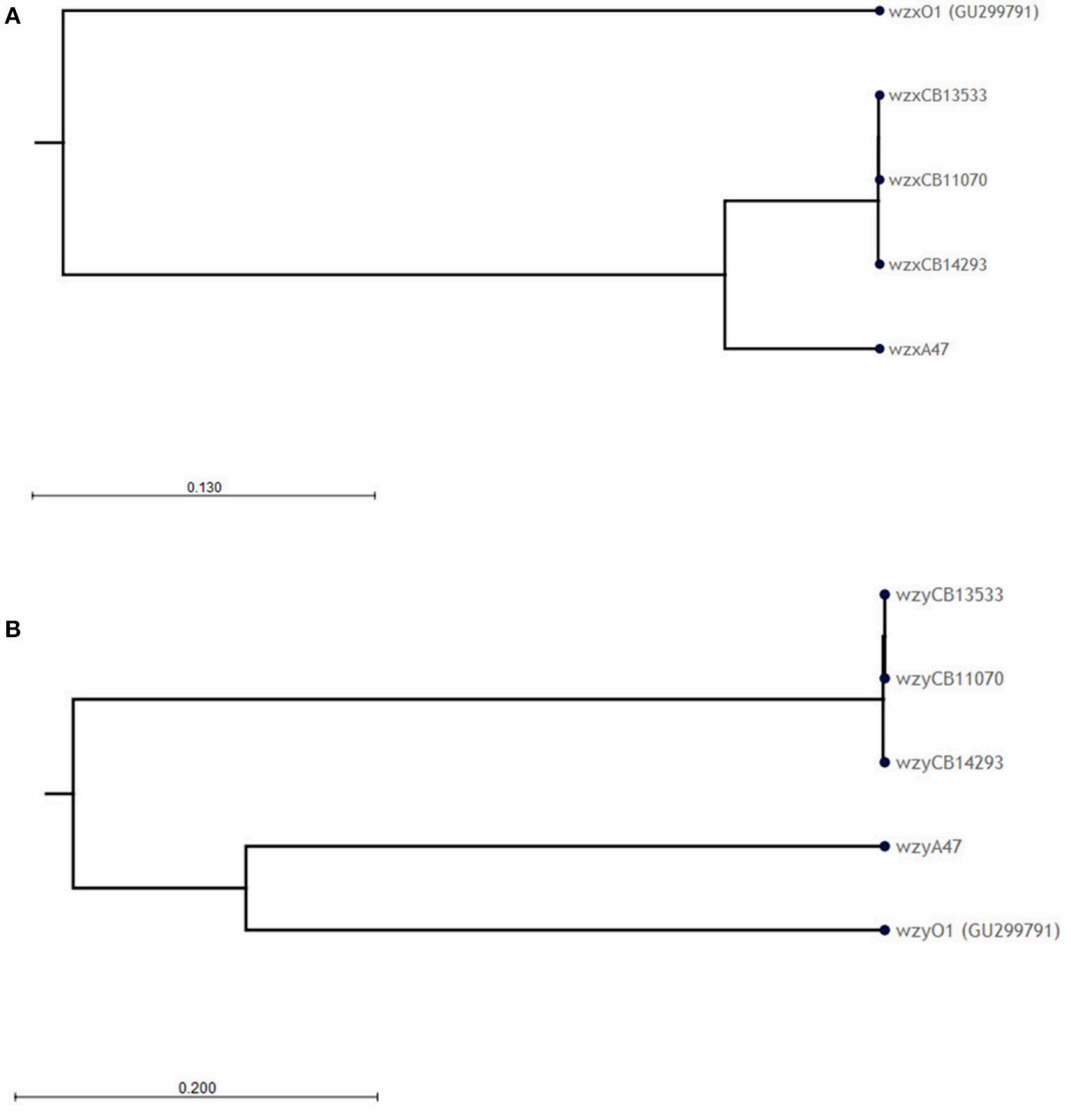
**Phylogenetic analysis of the (A)** *wzx* and **(B)** *wzy* nucleotide sequences of O1 strains. Phylogenetic trees were constructed using the CLC sequence viewer using the UPGMA algorithm. Reliability of the nodes is supported by bootstrap values > 70% (100 replicates).

The O-antigen gene clusters (O-AGC) of strains CB11070, CB13533, and CB14293 are approximately 10 kb in length; they all contain 10 ORFs between the *galF* and *gnd* genes which all have the same transcriptional direction from *galF* to *gnd* (Figure 1, Supplementary Table S1). The overall structure of their O-AGC is similar to that of G1632 with the same genes in the same order (Figure 1). The nucleotide sequence of the *wzx* and *wzy* genes of the 3 strains are closely related (99.8–100% identity; Figure 2). All the other genes from the cluster are similarly related with an overall similarity between the three clusters of 82.5–94.1% (Supplementary Data S2). All sequences, including *wzx* and *wzy*, are however distantly related to those of the O1 reference strain G1632 (41–55% identity). Besides the *rmlBDAC* genes, which are fairly common genes among various serogroups (Iguchi et al., 2015a; DebRoy et al., 2016), the other ORFs, including the *wzx* and *wzy* gene sequences, do not have any significant similarity in the non-redundant nucleotide database of Genbank (Supplementary Table S3).

The O-AGC of strain A47 is different from the other strains (Figure 1). It contains 12 ORFS between *galF* and *gnd* in the same transcriptional order from *galF* to *gnd* (Supplementary Table S1). It is approximately 16 kb in length and has an overall structure different from G1632 reference sequence. The 5′-end of the cluster is similar to the other O1 strains, containing the *rmlBDAC* gene cluster followed by the *wzx* gene, the *mnaA* gene (encoding a UDP-N-acetylglucosamine 2-epimerase) and a glycosyl transferase. However, the second half of the cluster harbors a different set of glycosyl transferase genes. The nucleotide sequence of the *wzx* gene of strain A47 is somewhat related to that of strains CB11070, CB13533, and CB14293 as they share 89% identity (89.1–89.2%). As expected, it is distantly related to that of G1632 (54.4% identity) and no significant similarity was found in the nucleotide database of Genbank (Supplementary Table S3). Similarly, the *mnaA* gene and glycosyl transferase (PROKKA_00007) following *wzx* do not reveal any significant similarity in the nucleotide database of Genbank (Supplementary Table S1). The nucleotide sequence of the *wzy* gene however is very different from that of G1632 (44.7% identity) and to those of the 3 other strains (48% identity). A blastn search indicates that it is 89% identical to the *wzy* gene of O10:K5:H4 strain Bi8337-41. Interestingly, a blast analysis of the glycosyl transferase genes surrounding *wzy* (PROKKA_00006, PROKKA_00004, and PROKKA_00003) shows 90–94% identity to the O10 strain Bi8337-41 O-AGC (Supplementary Table S3). The last ORF of A47 O-AGC (PROKKA_00002) shows 91% identity to the *wekS* gene of O2 strain G1674 O-AGC (Supplementary Table S3).

### Analysis of the O-antigen encoding genes in *E. coli* O2 strains

We confirmed the published sequence of O50 reference strain U18-41 by *de novo* sequencing. The O-AGC for serogroups O2 and O50 are over 99% identical (Iguchi et al., 2015a; DebRoy et al., 2016). An alignment of O2 and O50 O-AGC sequences show that they differ by less than 10 SNPs with only 2 SNPs apparently differentiating O2 and O50 (not due to inter-individual variations or sequencing errors) which could thus be considered as a single serogroup (Supplementary Table S4).

A qPCR was developed for the *E. coli* O2/O50*wzx* gene derived from the sequence of the *E. coli* O2 strain G1674 (Li et al., 2010; GenBank Accession GU299792.1; Table 1). This PCR was tested on an initial panel of 23 *E. coli* O2 strains and reacted only with 13 of these (Table 4, see below). The O2 antigen encoding genes of two non-reacting strains, CB11127 (O2:H49) and CB15123 (O2:H40), were analyzed by nucleotide sequencing.

**Table 4:** **Investigation of ***E. coli*** O2 strains by qPCR with O2 and K1 specific assays**.

**Serotype**	**Source, origin**	**References**	**Stx**	**Nos of strains**	**wzxO2**	**wzxO2-2**	**wzxO2-3**	***neuBK1***
O2:K1:H6	Infant feces, Germany	Wullenweber et al., 1993; Bettelheim et al., 2003	−	1	+	−	−	+
O2:K1:H7	Infant feces, Germany	Wullenweber et al., 1993; Bettelheim et al., 2003	−	1	+	−	−	+
O2: H1	Calf feces, Germany	This work, 2010	−	1	+	−	−	−
O2:K1:H4	Infant feces, Germany	Wullenweber et al., 1993; Bettelheim et al., 2003	−	1	+	−	−	+
O2:K5:H4	Infant feces, Germany	Wullenweber et al., 1993; Bettelheim et al., 2003	−	2	+	−	−	−
O2:H25	See footnote a	See footnote a	+	3	+	−	−	−
O2:H32	Wild boar, Germany	This work, 2011	stx2	1	+	−	−	−
O2:H27	Cattle feces, Germany	This work, 2011	stx2	2	+	−	−	−
O2:K2:H1	Infant feces, Germany	Wullenweber et al., 1993; Bettelheim et al., 2003	−	1	+	−	−	−
O2:H40	See footnote b	See footnote b	−	3	−	−	+	−
O2:H34	Cat feces, Brazil	Morato et al., 2009	−	1	−	+	−	−
O2:H49	Pig feces, Switzerland	Fröhlicher et al., 2008	−	3	−	+	−	−
O2:H29	Cattle feces	Martin and Beutin, 2011	stx2	2	−	+	−	−
O2:H8	Sheep feces, Germany	Krause et al., 2005	−	1	−	−	+	−

a *One stx2-positive O2:H25 strain was isolated from calf feces (Germany, 2011). One stx2-positive O2:H25 strain is a stx2g reference strain (Scheutz et al., 2012). The only stx1-positive O2:H25 strain was isolated from cattle (Beutin et al., 1993)*.

b *O2:H40 strains were isolated from pig and sheep feces, (2007) (Fröhlicher et al., 2008) and chicken meat (this work, 2013)*.

**Figure 3:**
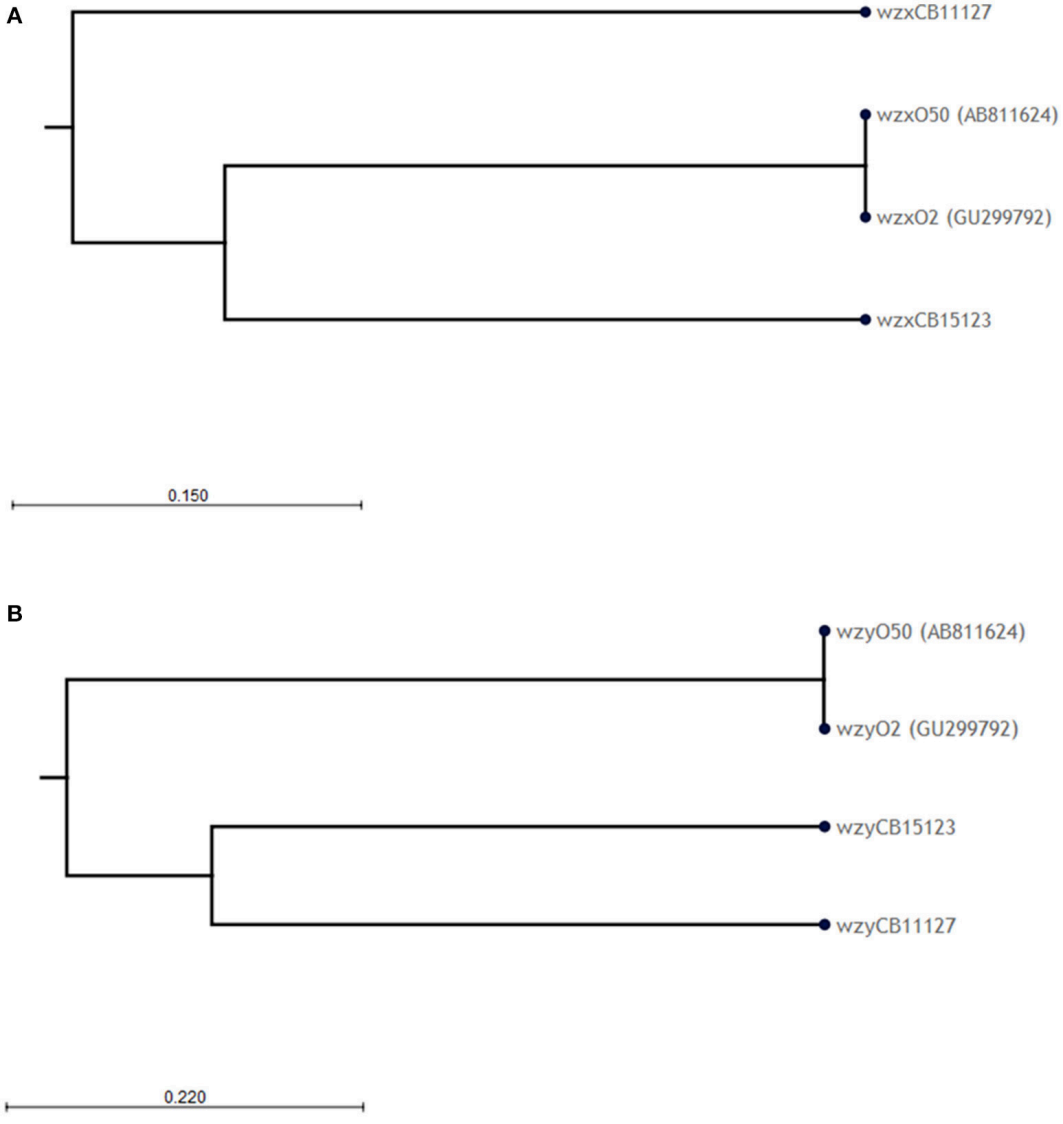
**Phylogenetic analysis of the (A)** *wzx* and **(B)** *wzy* nucleotide sequences of O2/O50 strains. Phylogenetic trees were constructed using the CLC sequence viewer using the UPGMA algorithm. Reliability of the nodes is supported by bootstrap values of 100% (100 replicates).

The O-AGC of both strains are different from each other and from the reference sequence G1674 (Figure 1). The O-AGC of strains CB11127 and CB15123 are approximately 15 kb in length and contain 14 and 15 ORFs between *galF* and *gnd* in the same transcriptional order from *galF* to *gnd*, respectively (Supplementary Table S1). The nucleotide sequence of the *wzx* and *wzy* genes of both strains have only 37.7–60.2% identity to each other and to the O2 and O50 reference strains (Figure 3). While O2 reference sequence G1674, O50 reference sequence U18-41 and CB15123 possess the *rmlBDAC* genes, CB11127 is missing the *rmlD* and *rmlC* genes. Both CB11127 and CB15123 also harbor the extra *manB* and *manC* genes of the GDP-mannose pathway and different sets of putative glycosyl transferases. Transposases are inserted between the *manC* and *manB* genes of CB11127 without any apparent gene disruption. Blast analyses of CB11127 ORFs indicate that most of them (including *wzx* and *wzy*) are significantly similar to a single sequence in Genbank nucleotide collection corresponding to strain FHI98 (strain of unknown serotype isolated from human feces in 2011) (Supplementary Table S3). Besides the *rmlBDA* genes which are found in the O-AGC of multiple isolates of various serogroups, blastn analyses of CB15123 do not find any significantly similar sequences for most of its ORFs (including *wzx* and *wzy*) in Genbank nucleotide collection (Supplementary Table S3).

### Development and evaluation of qPCR assays for identification of *E. coli* O1, O2, and K1 strains

Major differences between O1A and O1non-A strains were found in their corresponding *wzx* and *wzy* genes which are the principal targets used for molecular O-typing of *E. coli* (Iguchi et al., 2015a; Joensen et al., 2015). Certain sequence alterations in these two genes could be attributed to *E. coli* O1A and O1non-A strains, respectively, and qPCR assays for their specific detection were developed (Table 1). When tested with an initial panel of 36 well characterized O1 strains, the qPCR assay derived from the sequence of the ExPEC O1A strain U5-41 (wzyO1A) detected all O1 strains belonging to H-types H6, H7, H10, H31, H34, and H42. The qPCR assay derived from the STEC O1:H20 strain CB13533 (wzxO1non-A) detected all O1 strains belonging to H-types H12, H19, H20, and H33 (Table 3). We have tested the qPCRs developed for detection of O1A type and O1 non-A strains for reaction with the reference strains for serogroups O1-O186. None of the reference strains belonging to other O-groups than O1 reacted with the wzyO1A or with the wzxO1non-A qPCR assays. Additionally, they were tested against a panel of 153 strains (Table 5), including 130 strains of serogroups other than O1, belonging to 43 different serogroups. None of the strains belonging to O-groups other than O1 reacted with the wzyO1A or with the wzxO1non-A qPCR assays. The wzyO1A qPCR assay reacted only with strains of serotype O1:K1:H7 (*n* = 17), while the wzxO1non-A qPCR assay reacted only with strains of serotype O1:H12 (*n* = 1), O1:H20 (*n* = 3), O1:H21 (*n* = 1), and O1:H33 (*n* = 1). Thus, the qPCR detection method was found to be far more specific for identification of *E. coli* O1 strains than conventional O-serotyping. Moreover, the qPCR assays used here discerned clearly between *E. coli* O1A and O1non-A type strains, respectively.

**Table 5:** *****E. coli*** strains tested by real-time PCR**.

**Group (n° of strains)**	**Serotypes (n° of strains)**
O1 (*n* = 23)	O1:K1:H7 (*n* = 17); O1:[H21]; O1:H20 (*n* = 3); O1:H33; O1:H12
O2 (*n* = 38)	O2:H32; O2:H25 (*n* = 4); O2:K5:H4 (*n* = 2); O2:K1:H4; O2:K2:H1; O2:H1; O2:K5:H1; O2:H27 (*n* = 3); O2:K1:H6; O2:K1:H7; O2:K1:H4 (*n* = 2); O2:K1:H1 (*n* = 2); O2:K1:H5 (*n* = 3); O2:K1:H6 (*n* = 3); O2:K1:H48; O2:H34; O2:H49 (*n* = 3), O2:H29 (*n* = 2); O2:H8; O2:H40 (*n* = 4)
K1 (non O1, non O2) (*n* = 4)	ONT:K1:H4; [O134]:K1:HNT; [O166]:K1:H7; [O13/O135/O129]:K1:H4
Other (*n* = 88)	O4:H5; O4:H16; O6 (*n* = 2); O6:H4; O6:H10; O6:H34 (*n* = 2); O7:H4; O8:H45; O9:H12 (*n* = 2); O15:H2; O15:H11; O15:H16; O15:H21; O17/77:H41; O20:H9; O20:H30; O20:H33; O20:NM; O23:H15; O28:H28; O33:H12; O40:H6; O41:H7; O45:H20; O45:H31; O51:H49; O55:H6; O55:H7 (*n* = 3); O55:H19; O55:H21; O55:[H51]; O63:H6; O68:H12; O76:H41; O86:H34; O91:H28; O103:H2; O110:H28; O113:H6 (*n* = 2); O114:H49; O118:H2; O118:H5; O118:H8; O118:H12; O118:H16; O121:H45; O125:H6; O126:H6; O127:H6; O128:H2; O128:H8; O132:H1; O132:H34; O136:H2; O136:H12; O136:NM; O139:H1; O139:H4; O139:H19; O139:H+; O139; O142:H34; O145:H28; O145:H34; O153:H7; O153:H12; O153:H14; O153:H21; O153:H25; O157:H7 (*n* = 4); O157:H12 (*n* = 2); O157:H15; O157:H45; O179:H31; O181:H49; O186:[H45]; OX185:H28; Or:H12

*For each serotype, n = 1 unless otherwise stated. Serotypes between brackets were determined by sequencing*.

The divergence between the *wzx* sequences of strains U9-41 (O2:K1:H4), CB11127 (O2:H49), and CB15123 (O2:H40) allowed us to design three qPCR assays, targeting the O2/O50 and two O2-like serogroups respectively (Table 1). When tested with an initial panel of 23 well characterized O2 strains, the qPCR assay derived from the sequence of the ExPEC O2 strain U9-41 (wzxO2) detected all O2 strains belonging to H-types H1, H4, H6, H7, and H25. The qPCR assay derived from the O2:H49 strain CB11127 (wzxO2-2) detected the O2 strains belonging to H-type H49, H34, and H29. The qPCR assay derived from the O2:H40 strain CB15123 (wzxO2-3) detected all O2:H40 strains and the O2:H8 strain (Table 4). The wzxO2, wzxO2-2, and wzxO2-3 qPCRs did not react with any of the O1-O186 reference strains except for wzxO2 qPCR test which reacted with reference strains for serogroups O2 and O50. Additionally, when tested against the panel of 153 strains (Table 5), including 115 strains of serogroups other than O2, none of the strains belonging to O-groups other than O2 reacted with the wzxO2, the wzxO2-2 or with the wzxO2-3 qPCR assays. The wzxO2 qPCR assay reacted only with ExPEC and STEC strains of serotype O2:H1 (*n* = 2), O2:K5:H1 (*n* = 1), O2:K1:H1 (*n* = 2), O2:K5:H4 (*n* = 2), O2:K1:H4 (*n* = 3), O2:K1:H5 (*n* = 3), O2:K1:H6 (*n* = 4), O2:K1:H7 (*n* = 1), O2:H25 (*n* = 4), O2:H27 (*n* = 3), O2:H32 (*n* = 1) and O2:K1:H48 (*n* = 1). The wzxO2-2 qPCR assay reacted with EPEC and STEC strains of serotype O2:H34 (*n* = 1), O2:H49 (*n* = 3), and O2:H29 (*n* = 2). The wzxO2-3 qPCR reacted only with EPEC strains of serotype O2:H8 (*n* = 1) and O2:H40 (*n* = 4).

The specificity of the *neuB* qPCR for indicating strains producing the K1 antigen was tested on the serotype reference strains O1-O186 which have been analyzed for their capsular antigens (Orskov and Orskov, 1984; Orskov et al., 1984; Scheutz et al., 2004; Joensen et al., 2015). None of the strains except those carrying the K1 antigen (U5-41, U9-41, Bi7509-41, F11119-41, and H61) reacted with the *neuB* qPCR. The *neuB* qPCR was further evaluated on *E. coli* K1 strains belonging to different O-groups which were from clinical samples and from stool of healthy infants (Wullenweber et al., 1993). In the group of *E. coli* O1 strains, the K1 antigen gene was only detected in O1:K1:H7 strains isolated from humans and poultry (Table 3). In the group of *E. coli* O2 strains, the K1 antigen gene was detected in O2:K1:H1, O2:K1:H4, O2:K1:H5, O2:K1:H6, O2:K1:H7, O2:K1:H9, and O2:K1:H48, from humans and animals (Table 4). Among the other strains of serogroups other than O1 and O2, only four ExPEC strains isolated from humans (ONT:K1:H4, O134:K1:HNT, O166:K1:H7, and O13/O135/O129:K1:H4) reacted with the K1 qPCR assay (Table 5). These four strains were previously found K1-positive by latex agglutination.

## Discussion

*Escherichia coli* strains of serogroups O1 and O2 belong to the most frequently isolated *E. coli* types from humans (Orskov and Orskov, 1992; Olesen et al., 1995; Bettelheim et al., 2003; Ciesielczuk et al., 2016) and also have a broad reservoir in pets and domestic animals (Moulin-Schouleur et al., 2007; Dziva and Stevens, 2008; Ewers et al., 2014). Because of their incidence, clinical importance and type diversity, a reliable identification of *E. coli* O1 and O2 serogroups together with the frequently associated K1 capsule is important for diagnosis and source attribution of *E. coli* infections in humans and animals. Detection of *E. coli* O1 and O2 strains by conventional serotyping with O-antigen specific rabbit antisera is hampered by cross reactions that were found between *E. coli* O1 and O2 and also with many other *E. coli* O-serogroups (Orskov and Orskov, 1984; Edwards and Ewing, 1986). Detection of the K1 capsule is made either by serotyping or by lysotyping with K1-specific bacteriophages (Gross et al., 1977; Orskov et al., 1977). Both methods are costly and rather difficult to introduce in routine clinical diagnosis and would be advantageously replaced by molecular methods.

The LPS of *E. coli* O1 strains exhibit some structural variations which cross-react in conventional serotyping assays. The common epitope between the O1 variants contains an *N*-acetylmannosamine residue branched on an *L*-rhamnose residue (Gupta et al., 1992). Moreover, *E. coli* O1 antisera were reported to show multiple cross-reactions with other O-serogroups. With the O1 antiserum used here for serotyping, serological cross-reactions with reference strains for O-serogroups O2, O10, O50, O53, O107, O115, O117, O148, O149, and O154 were observed, corresponding to previously published findings (Orskov and Orskov, 1984; Edwards and Ewing, 1986). All of these O-groups possess the *rmlBDAC* genes (Iguchi et al., 2015a; DebRoy et al., 2016) involved in the synthesis of *L*-rhamnose residues which must be part of their antigenic epitope and may be the cause of the serological cross-reactions. Similarly, O2 antisera can show cross-reactions with serogroups O1, O2/O50, O53, O74, and O117 (Orskov et al., 1977). The O2 antiserum used here agglutinated strains belonging to these O-groups as well as O184 which was not in the serotyping scheme published by Orskov in 1977 (Orskov et al., 1977). The molecular basis of these serological cross-reactions is not clear as the O-AGC of these serogroups exhibit few common features.

In an effort to build a complete molecular serotyping scheme for *E. coli* we sought to develop qPCR assays for the specific detection of O1, O2 LPS and the K1 capsule. Based on the nucleotide sequence of the *wzy* gene of the O1 reference strain G1632 (U5-41) we developed a qPCR assay for specific detection of O1 strains. While this assay appeared highly specific, as it did not cross react with any other serogroups, several strains identified as O1 with the seroagglutination failed to react. In the same way, a highly specific qPCR assay developed based on the *wzx* gene of the O2 reference strain G1674 failed to react with several strains identified as O2 with the seroagglutination. The qPCR assay developed for specific detection of the *neuB* gene of K1 strains proved 100% sensitive and 100% specific.

Analysis of the O-AGC of non-reacting O1 and O2 strains revealed significant nucleotide sequence divergences. The O-AGC in the non-reacting O1 strains CB11070, CB13533, and CB14293 appears to be a variant of the one in O1A reference strain G1632. The O1A polysaccharide contains five sugar residues: 3 *L*-rhamnose residues, an *N*-acetylglucosamine residue and an *N*-Acetyl-*D*-mannosamine residue. The O1B/O1C variants contain two *L*-rhamnose residues, an *N*-acetylglucosamine residue, an *N*-Acetyl-*D*-mannosamine residue and a *D*-Galactose residue (Moll et al., 1986; Gupta et al., 1992). Strains CB11070, CB13533, and CB14293 all apparently conserved the ability to synthesize *L*-rhamnose residues, *N*-acetylglucosamine residues and *N*-Acetyl-*D*-mannosamine residues. The presence of a *D*-Galactose residue in these strains cannot be inferred from the O-AGC sequence. Presence of other residues whose biosynthesis genes are located outside of the O-AGC cannot be ruled out. Indeed, genes involved in the biosynthesis of the nucleotide precursors of common sugars are usually located outside O-antigen gene clusters (Stenutz et al., 2006). Additional LPS-modifying enzymes can also be found elsewhere in the chromosome (Wang et al., 2002), especially in bacteriophages (Allison and Verma, 2000; Perry et al., 2009). The differences in genetic sequence of the whole cluster suggest a clonal divergence and parallel evolution of O1A and O1nonA strains. The genetic structure of the O-AGC of strain A47 appears to be a mosaic structure containing three modules. The first module (in the transcriptional orientation), containing seven genes including *wzx*, derives from the same O1 variant. The second module, containing four genes including *wzy*, is related to the O10 O-AGC. The third module contains a single gene derived from the O2-OAGC. This mosaic structure suggests a recombination between the O-AGC of strains of serogroups O1, O10 and O2. Generation of new serogroup variants through recombination of parts of the O-AGC has already been reported for serogroup O9 (Sugiyama et al., 1998) and could be used to generate new epitopes and evade detection by the immune system of the host.

The O-AGC found in O2 strains CB11127 and CB15123 constitute two new O2-like serogroups. The genetic structure of the O-AGCs in strains CB11127 and CB15123 are different from that of the O2 reference strain G1674 suggesting different LPS structures. The O2 polysaccharide contains three L-rhamnose residues, an *N*-acetylglucosamine residue, and an N-acetyl-D-galactosamine (Jansson et al., 1987). Strain CB11127 most likely lacks the *L*-rhamnose residues as the absence of the *rmlC* and *rmlD* genes prevents full transformation of glucose-1-phosphate into dTDP-*L*-Rhamnose. Instead, presence of the *manB* and *manC* genes suggests the presence of residue(s) derived from GDP-*D*-Mannose. Presence of *rmlA, rmlB, fdtA, fdtB*, and *fdtC* suggest that the N-acetyl-D-galactosamine residue is conserved. Strain CB15123 conserved both the ability to synthesize *L*-rhamnose residues and N-acetyl-*D*-galactosamine residues and also acquired the GDP-*D*-Mannose pathway. Once again, presence of other residues whose biosynthesis genes are located outside of the O-AGC cannot be ruled out.

Based on the nucleotide sequence of the new O1-variant and the two O2-like serogroups, specific qPCR assays were developed, which proved to be 100% sensitive and 100% specific, and able to clearly distinguish the different genetic variants identified within each serogroup.

Overall, the various qPCR assays designed in this study can be used for detection and rapid identification of the different types of *E. coli* strains of serogroups O1 and O2. They provide a significantly improved alternative to classical O-serotyping by their high specificity and sensitivity to detect virtually all types of O1 and O2 strains and their respective associated variants. They are therefore potentially useful for direct identification of clinically important O1 and O2 strains in food and clinical specimens. These different qPCR assays can thus serve as tools for epidemiological investigations and source attribution of human infections. Molecular typing by use of specific qPCRs provides a more specific detection of clinically important O1 and O2 ExPEC strains. Ambiguous results obtained by serotyping such as cross-reactions with other *E. coli* O-groups and agglutination reactions with O-rough strains are avoided. Moreover, the qPCR approach described in this study provides a better discrimination between O1 and O2 ExPEC and non-ExPEC strains. In combination with the detection of the *neuB* gene (K1 capsule), life-threatening O1 and O2 ExPEC infections in risk groups such as newborn infants can be more rapidly identified. Asymptomatic carriers, animals and food contaminated with O1 and O2 ExPEC strains can be more easily identified and the spread of pathogens to human risk groups (new born-infants, elderly, and intensive-care patients) can thus be prevented. The qPCR detection of ExPEC O1 and O2 strains is a reliable and easy approach to elucidate the significance of zoonotic and food-borne infection of humans with ExPEC O1 and O2 strains in epidemiological investigations.

## Author contributions

Conceived and designed the experiments: LB, SD, and PF. Performed the experiments: SD, LB, PM, AF, and SB. Analyzed the data: SD, LB, PM, AF, SB, and PF. Contributed reagents/materials/analysis tools: SD, LB, PM, SB, and PF. Wrote the paper: SD, LB, and PF. Critical revision of the paper for important intellectual content: SD, LB, PM, AF, SB, and PF.

### Conflict of interest statement

The authors declare that the research was conducted in the absence of any commercial or financial relationships that could be construed as a potential conflict of interest.
