# Supplementary material for: The Escherichia coli Serogroup O1 and O2 Lipopolysaccharides Are Encoded by Multiple O-antigen Gene Clusters
Source: Front Cell Infect Microbiol. 2017 Feb 7;7:30. doi: 10.3389/fcimb.2017.00030 (PMC5293828; doi:10.3389/fcimb.2017.00030)
Supplement: Supplementary file 4 [file Table4.docx]

**Supplementary Table S4**: Multiple sequences alignment of the O-AGC of the O50 and O2 strains. Strains are named by their accession number, except for U18-41-new which represents the U18-41 as sequenced in this study (accession KY115227). Only the top sequence is indicated in full. In the other sequences only alternate bases are indicated. Identical bases are indicated by “.”. Sequences were truncated at the 5’-end such as at least two sequences had the same starting point. Numbering is according to the top sequence input.

10 20 30 40 50 60 70 80

....|....|....|....|....|....|....|....|....|....|....|....|....|....|....|....|

**U18-41-new** **TGGTAGCTGT-AAGCCAAGGGCGGTAGCGTGCATTAATACTTCTATTAATCAAATCTCGAGCAGTCTATTTCACAGTATG**

**AB811624.1** **..........T......G..............................................................**

**GU299792.1** **AT........-.....................................................................**

**EU549863.1** **-------------------------------------...........--TC............................**

90 100 110 120 130 140 150 160

....|....|....|....|....|....|....|....|....|....|....|....|....|....|....|....|

**U18-41-new** **CTCTCTGGCTATATGGAATAAAAAAGTGAAGATACTTGTTACTGGTGGCGCAGGCTTTATTGGTTCTGCTGTTGTTCGTC**

**AB811624.1** **................................................................................**

**GU299792.1** **................................................................................**

**EU549863.1** **................................................................................**

170 180 190 200 210 220 230 240

....|....|....|....|....|....|....|....|....|....|....|....|....|....|....|....|

**U18-41-new** **ACATAATAAATAATACGCAAGATAGTGTTGTTAATGTCGATAAATTAACATACGCCGGAAACCTGGAATCGCTCGCTGAA**

**AB811624.1** **................................................................................**

**GU299792.1** **................................................................................**

**EU549863.1** **................................................................................**

250 260 270 280 290 300 310 320

....|....|....|....|....|....|....|....|....|....|....|....|....|....|....|....|

**U18-41-new** **GTTTCTGATTCTGAGCGTTATTCATTTGAGCATGCAGATATCTGCGATGCCGAAGCGATGGCGCGTATTTTTTCACAACA**

**AB811624.1** **................................................................................**

**GU299792.1** **................................................................................**

**EU549863.1** **................................................................................**

330 340 350 360 370 380 390 400

....|....|....|....|....|....|....|....|....|....|....|....|....|....|....|....|

**U18-41-new** **CCAGCCAGATGCGGTGATGCACCTTGCGGCTGAGAGCCACGTTGACCGCTCAATAACTGGCCCGGCGGCATTCATTGAAA**

**AB811624.1** **................................................................................**

**GU299792.1** **................................................................................**

**EU549863.1** **................................................................................**

410 420 430 440 450 460 470 480

....|....|....|....|....|....|....|....|....|....|....|....|....|....|....|....|

**U18-41-new** **CTAATATTGTAGGTACTTACGTACTTTTAGAAGCTGCGCGCAATTATTGGTCTGGTCTGGATGATGAAAAGAAAAAAAAC**

**AB811624.1** **................................................................................**

**GU299792.1** **................................................................................**

**EU549863.1** **................................................................................**

490 500 510 520 530 540 550 560

....|....|....|....|....|....|....|....|....|....|....|....|....|....|....|....|

**U18-41-new** **TTCCGCTTTCATCATATTTCTACTGATGAAGTATATGGTGACTTACCCCATCCGGATGAAGTAAATAATAACGAAGCGTT**

**AB811624.1** **................................................................................**

**GU299792.1** **.........T......................................................................**

**EU549863.1** **................................................................................**

570 580 590 600 610 620 630 640

....|....|....|....|....|....|....|....|....|....|....|....|....|....|....|....|

**U18-41-new** **ACCGCTATTTACGGAAACGACAGCCTACGCGCCAAGTAGCCCGTATTCTGCTTCTAAAGCTTCCAGCGATCATTTGGTTC**

**AB811624.1** **................................................................................**

**GU299792.1** **................................................................................**

**EU549863.1** **................................................................................**

650 660 670 680 690 700 710 720

....|....|....|....|....|....|....|....|....|....|....|....|....|....|....|....|

**U18-41-new** **GCGCATGGAAACGTACGTATGGTTTACCGACCATTGTGACTAATTGCTCGAACAACTATGGTCCGTATCACTTCCCGGAA**

**AB811624.1** **................................................................................**

**GU299792.1** **................................................................................**

**EU549863.1** **................................................................................**

730 740 750 760 770 780 790 800

....|....|....|....|....|....|....|....|....|....|....|....|....|....|....|....|

**U18-41-new** **AAGCTTATTCCATTGGTTATTCTTAATGCACTGGAAGGTAAGGCATTACCTATTTATGGCAAAGGGGATCAAATTCGCGA**

**AB811624.1** **................................................................................**

**GU299792.1** **................................................................................**

**EU549863.1** **................................................................................**

810 820 830 840 850 860 870 880

....|....|....|....|....|....|....|....|....|....|....|....|....|....|....|....|

**U18-41-new** **CTGGTTGTATGTAGAGGATCATGCTCGTGCGTTATATACCGTCGTAACCGAAGGTAAAGCGGGTGAAACTTATAACATTG**

**AB811624.1** **................................................................................**

**GU299792.1** **................................................................................**

**EU549863.1** **................................................................................**

890 900 910 920 930 940 950 960

....|....|....|....|....|....|....|....|....|....|....|....|....|....|....|....|

**U18-41-new** **GCGGACACAACGAAAAGAAAAACATCGATGTTGTGCTGACTATTTGTGATTTGTTGGATGAGATTGTACCGAAAGAGAAA**

**AB811624.1** **................................................................................**

**GU299792.1** **................................................................................**

**EU549863.1** **................................................................................**

970 980 990 1000 1010 1020 1030 1040

....|....|....|....|....|....|....|....|....|....|....|....|....|....|....|....|

**U18-41-new** **TCTTATCGTGAGCAAATTACTTATGTTGCTGATCGCCCAGGGCATGATCGCCGTTATGCAATTGATGCCGATAAAATTAG**

**AB811624.1** **................................................................................**

**GU299792.1** **................................................................................**

**EU549863.1** **................................................................................**

1050 1060 1070 1080 1090 1100 1110 1120

....|....|....|....|....|....|....|....|....|....|....|....|....|....|....|....|

**U18-41-new** **CCGCGAATTGGGCTGGAAACCACAGGAAACGTTTGAGAGCGGGATTCGCAAAACGGTGGAATGGTATCTGGCTAATACAA**

**AB811624.1** **................................................................................**

**GU299792.1** **........................................T.......................................**

**EU549863.1** **................................................................................**

1130 1140 1150 1160 1170 1180 1190 1200

....|....|....|....|....|....|....|....|....|....|....|....|....|....|....|....|

**U18-41-new** **ATTGGGTTGAGAATGTGAAAAGCGGTGCTTATCAGTCATGGATCGAACAAAACTATGAGGGCCGTCAGTAATGAATATCC**

**AB811624.1** **................................................................................**

**GU299792.1** **................................................................................**

**EU549863.1** **................................................................................**

1210 1220 1230 1240 1250 1260 1270 1280

....|....|....|....|....|....|....|....|....|....|....|....|....|....|....|....|

**U18-41-new** **TGCTTTTCGGCAAAACAGGGCAGGTGGGTTGGGAACTGCAGCGTGCTCTGGCGCCGCTGGGTAATCTGATCGCTCTTGAT**

**AB811624.1** **................................................................................**

**GU299792.1** **................................................................................**

**EU549863.1** **................................................................................**

1290 1300 1310 1320 1330 1340 1350 1360

....|....|....|....|....|....|....|....|....|....|....|....|....|....|....|....|

**U18-41-new** **GTTCACTCCACTAATTATTGTGGAGATTTCAGCAACCCCGAAGGTGTGGCAGAAACCGTCAAAAAAATTCGTCCTGACGT**

**AB811624.1** **................................................................................**

**GU299792.1** **................................................................................**

**EU549863.1** **................................................................................**

1370 1380 1390 1400 1410 1420 1430 1440

....|....|....|....|....|....|....|....|....|....|....|....|....|....|....|....|

**U18-41-new** **TATTGTTAATGCTGCTGCTCACACTGCAGTAGATAAAGCAGAATCAGAACCGGATTTCGCACAATTACTTAACGCGACAA**

**AB811624.1** **................................................................................**

**GU299792.1** **................................................................................**

**EU549863.1** **................................................................................**

1450 1460 1470 1480 1490 1500 1510 1520

....|....|....|....|....|....|....|....|....|....|....|....|....|....|....|....|

**U18-41-new** **GCGTCGAAGCGATTGCAAAAGCTGCTAATGAAGTCGGGGCCTGGGTTATACACTACTCTACTGATTATGTTTTCCCAGGC**

**AB811624.1** **................................................................................**

**GU299792.1** **................................................................................**

**EU549863.1** **................................................................................**

1530 1540 1550 1560 1570 1580 1590 1600

....|....|....|....|....|....|....|....|....|....|....|....|....|....|....|....|

**U18-41-new** **AGTGGTGACGCGCCATGGCTGGAAACGGATGCAACAGCACCGCTAAATGTTTACGGTGAAACAAAATTAGCTGGGGAAAA**

**AB811624.1** **................................................................................**

**GU299792.1** **................................................................................**

**EU549863.1** **................................................................................**

1610 1620 1630 1640 1650 1660 1670 1680

....|....|....|....|....|....|....|....|....|....|....|....|....|....|....|....|

**U18-41-new** **GGCATTACAAGAACATTGCGCAAAGCATCTTATTTTCCGTACCAGCTGGGTATACGCTGGTAAAGGAAATAACTTTGCTA**

**AB811624.1** **................................................................................**

**GU299792.1** **................................................................................**

**EU549863.1** **................................................................................**

1690 1700 1710 1720 1730 1740 1750 1760

....|....|....|....|....|....|....|....|....|....|....|....|....|....|....|....|

**U18-41-new** **AAACGATGTTGCGTTTGGCAAAAGAACGCGAAGAACTGGCTGTGATAAACGATCAGTTTGGCGCACCAACAGGTGCTGAA**

**AB811624.1** **................................................................................**

**GU299792.1** **................................................................................**

**EU549863.1** **................................................................................**

1770 1780 1790 1800 1810 1820 1830 1840

....|....|....|....|....|....|....|....|....|....|....|....|....|....|....|....|

**U18-41-new** **TTGCTGGCTGATTGCACCGCTCATGCCATTCGCGTGGCATTAAAAAAACCAGAAGTCGCTGGCTTGTACCATCTGGTAGC**

**AB811624.1** **................................................................................**

**GU299792.1** **................................................................................**

**EU549863.1** **................................................................................**

1850 1860 1870 1880 1890 1900 1910 1920

....|....|....|....|....|....|....|....|....|....|....|....|....|....|....|....|

**U18-41-new** **AAGTGGCACAACAACCTGGCACGATTATGCTGCGCTGGTTTTTGAAGAGGCGCGCAAAGCAGGGATTAATCTTGCACTTA**

**AB811624.1** **................................................................................**

**GU299792.1** **................................................................................**

**EU549863.1** **................................................................................**

1930 1940 1950 1960 1970 1980 1990 2000

....|....|....|....|....|....|....|....|....|....|....|....|....|....|....|....|

**U18-41-new** **ACAAACTTAACGCCGTGCCAACAACGGCCTATCCCACACCAGCCCGTCGACCCCATAACTCTCGCCTCAATACAGAAAAG**

**AB811624.1** **................................................................................**

**GU299792.1** **................................................................................**

**EU549863.1** **................................................................................**

2010 2020 2030 2040 2050 2060 2070 2080

....|....|....|....|....|....|....|....|....|....|....|....|....|....|....|....|

**U18-41-new** **TTTCAGCAGAACTTTGCGCTTGTCTTGCCTGACTGGCAGGTGGGCGTGAAACGTATGCTCAACGAATTATTTACGACTAC**

**AB811624.1** **................................................................................**

**GU299792.1** **................................................................................**

**EU549863.1** **................................................................................**

2090 2100 2110 2120 2130 2140 2150 2160

....|....|....|....|....|....|....|....|....|....|....|....|....|....|....|....|

**U18-41-new** **GGCAATTTAACAAATTTTTGCATCTCGCTCATGATGCCAGAGCGGGATGAATTAAAAGGAATGGTGAAATGAAAACGCGT**

**AB811624.1** **................................................................................**

**GU299792.1** **................................................................................**

**EU549863.1** **................................................................................**

2170 2180 2190 2200 2210 2220 2230 2240

....|....|....|....|....|....|....|....|....|....|....|....|....|....|....|....|

**U18-41-new** **AAAGGTATTATTCTGGCTGGTGGTTCCGGCACTCGTCTTTATCCTGTGACGATGGCAGTGAGTAAACAATTGCTGCCGAT**

**AB811624.1** **................................................................................**

**GU299792.1** **................................................................................**

**EU549863.1** **................................................................................**

2250 2260 2270 2280 2290 2300 2310 2320

....|....|....|....|....|....|....|....|....|....|....|....|....|....|....|....|

**U18-41-new** **TTATGATAAGCCGATGATTTATTATCCGCTTTCAACGCTTATGTTAGCGGGTATTCGCGATATTCTTATTATTAGTACGC**

**AB811624.1** **................................................................................**

**GU299792.1** **................................................................................**

**EU549863.1** **................................................................................**

2330 2340 2350 2360 2370 2380 2390 2400

....|....|....|....|....|....|....|....|....|....|....|....|....|....|....|....|

**U18-41-new** **CACAGGATACACCGCGTTTCCAACAATTATTGGGGGACGGGAGCCAGTGGGGTCTTAATCTACAGTATAAAGTACAACCG**

**AB811624.1** **................................................................................**

**GU299792.1** **................................................................................**

**EU549863.1** **................................................................................**

2410 2420 2430 2440 2450 2460 2470 2480

....|....|....|....|....|....|....|....|....|....|....|....|....|....|....|....|

**U18-41-new** **AGTCCGGATGGCCTGGCGCAAGCGTTTATTATTGGCGAAGACTTTATTGGTGGTGATGATTGTGCACTCGTACTTGGCGA**

**AB811624.1** **................................................................................**

**GU299792.1** **................................................................................**

**EU549863.1** **................................................................................**

2490 2500 2510 2520 2530 2540 2550 2560

....|....|....|....|....|....|....|....|....|....|....|....|....|....|....|....|

**U18-41-new** **TAATATCTTCTATGGACACGACTTGCCGAAATTGATGGAAGCTGCTGTTAACAAAGAAAGCGGTGCAACGGTATTTGCTT**

**AB811624.1** **................................................................................**

**GU299792.1** **................................................................................**

**EU549863.1** **................................................................................**

2570 2580 2590 2600 2610 2620 2630 2640

....|....|....|....|....|....|....|....|....|....|....|....|....|....|....|....|

**U18-41-new** **ATCACGTTAATGATCCTGAACGCTATGGTGTCGTGGAGTTTGATAATAACGGTACGGCAATTAGCCTGGAAGAAAAACCG**

**AB811624.1** **................................................................................**

**GU299792.1** **................................................................................**

**EU549863.1** **................................................................................**

2650 2660 2670 2680 2690 2700 2710 2720

....|....|....|....|....|....|....|....|....|....|....|....|....|....|....|....|

**U18-41-new** **CTGGAGCCAAAAAGCAACTATGCGGTTACTGGGCTTTATTTCTATGACAATGACGTTGTGGAAATGGCTAAAAACCTTAA**

**AB811624.1** **................................................................................**

**GU299792.1** **................................................................................**

**EU549863.1** **................................................................................**

2730 2740 2750 2760 2770 2780 2790 2800

....|....|....|....|....|....|....|....|....|....|....|....|....|....|....|....|

**U18-41-new** **GCCTTCTGCCCGTGGCGAACTGGAAATTACCGATATTAACCGTATTTATATGGAACAAGGACGTTTGTCTGTAGCCATGA**

**AB811624.1** **................................................................................**

**GU299792.1** **................................................................................**

**EU549863.1** **................................................................................**

2810 2820 2830 2840 2850 2860 2870 2880

....|....|....|....|....|....|....|....|....|....|....|....|....|....|....|....|

**U18-41-new** **TGGGGCGTGGCTATGCATGGTTGGATACAGGGACGCATCAAAGCCTTATTGAAGCAAGTAACTTCATTGCAACAATTGAA**

**AB811624.1** **................................................................................**

**GU299792.1** **................................................................................**

**EU549863.1** **................................................................................**

2890 2900 2910 2920 2930 2940 2950 2960

....|....|....|....|....|....|....|....|....|....|....|....|....|....|....|....|

**U18-41-new** **GAGCGTCAGGGATTAAAGGTATCTTGCCCGGAAGAGATTGCTTACCGTAAAGGGTTTATTGATGCCGAGCAGGTGAAAGT**

**AB811624.1** **................................................................................**

**GU299792.1** **................................................................................**

**EU549863.1** **................................................................................**

2970 2980 2990 3000 3010 3020 3030 3040

....|....|....|....|....|....|....|....|....|....|....|....|....|....|....|....|

**U18-41-new** **ATTAGCCGAACCGCTTATCAAGAATCAATATGGTCAATATTTGCTGAAAATGATCAGCGAATAGTATATGGGAACTCAAT**

**AB811624.1** **................................................................................**

**GU299792.1** **................................................................................**

**EU549863.1** **................................................................................**

3050 3060 3070 3080 3090 3100 3110 3120

....|....|....|....|....|....|....|....|....|....|....|....|....|....|....|....|

**U18-41-new** **GATGGATATTAAATTAATCTCTTTGCAAAAACATGGGGATGAGCGCGGTGCATTAATTGCTCTTGAAGAGCAACGAAATA**

**AB811624.1** **................................................................................**

**GU299792.1** **................................................................................**

**EU549863.1** **................................................................................**

3130 3140 3150 3160 3170 3180 3190 3200

....|....|....|....|....|....|....|....|....|....|....|....|....|....|....|....|

**U18-41-new** **TACCTTTCGAAGTCAAAAGAATATATTACATACTTGAGACTCTTAATGGAGTAAGACGCGGATTTCATGCGCACAAGGTT**

**AB811624.1** **................................................................................**

**GU299792.1** **................................................................................**

**EU549863.1** **................................................................................**

3210 3220 3230 3240 3250 3260 3270 3280

....|....|....|....|....|....|....|....|....|....|....|....|....|....|....|....|

**U18-41-new** **ACTCGTCAGTTAGCTATTGTAGTCAAGGGAGCTTGTAAATTTCATCTGGATAATGGTAAAGAAACAAAGCAGGTGGAACT**

**AB811624.1** **................................................................................**

**GU299792.1** **................................................................................**

**EU549863.1** **................................................................................**

3290 3300 3310 3320 3330 3340 3350 3360

....|....|....|....|....|....|....|....|....|....|....|....|....|....|....|....|

**U18-41-new** **TAATGATCCAACAATTGCGTTGCTGATAGAACCCTATATATGGCATGAAATGTATGATTTTAGTGATGATTGTGTGCTGC**

**AB811624.1** **................................................................................**

**GU299792.1** **................................................................................**

**EU549863.1** **................................................................................**

3370 3380 3390 3400 3410 3420 3430 3440

....|....|....|....|....|....|....|....|....|....|....|....|....|....|....|....|

**U18-41-new** **TTGTAATTGCGGATGATTTCTATAAAGAGTCTGATTATATCCGCAATTATGATGATTTTATTAGAAGAGTAAATTCAATT**

**AB811624.1** **................................................................................**

**GU299792.1** **................................................................................**

**EU549863.1** **................................................................................**

3450 3460 3470 3480 3490 3500 3510 3520

....|....|....|....|....|....|....|....|....|....|....|....|....|....|....|....|

**U18-41-new** **GAGAATTCATAAGCTAAGTGACGTCCAGACAACATCAATTGGTGATGGAACAACTATCTGGCAGTTTGTTGTGATACTAA**

**AB811624.1** **................................................................................**

**GU299792.1** **................................................................................**

**EU549863.1** **................................................................................**

3530 3540 3550 3560 3570 3580 3590 3600

....|....|....|....|....|....|....|....|....|....|....|....|....|....|....|....|

**U18-41-new** **AAGGTGCTGTAATTGGTAATAATTGCAACATCTGTGCAAATACCTTAATTGAAAATAACGTTGTAATTGGTAACAATGTC**

**AB811624.1** **................................................................................**

**GU299792.1** **................................................................................**

**EU549863.1** **................................................................................**

3610 3620 3630 3640 3650 3660 3670 3680

....|....|....|....|....|....|....|....|....|....|....|....|....|....|....|....|

**U18-41-new** **ACAGTCAAAAGCGGTGTGTATATTTGGGATGGCGTTAAAATAGAGGATAATGTTTTTATTGGTCCTTGTGTAGCATTTAC**

**AB811624.1** **................................................................................**

**GU299792.1** **................................................................................**

**EU549863.1** **................................................................................**

3690 3700 3710 3720 3730 3740 3750 3760

....|....|....|....|....|....|....|....|....|....|....|....|....|....|....|....|

**U18-41-new** **AAATGATAAGTATCCTCGCTCTAAAGTCTATCCTGATGAATTTTTGCAAACAATAATACGCAAAGGAGCATCAATAGGTG**

**AB811624.1** **................................................................................**

**GU299792.1** **................................................................................**

**EU549863.1** **................................................................................**

3770 3780 3790 3800 3810 3820 3830 3840

....|....|....|....|....|....|....|....|....|....|....|....|....|....|....|....|

**U18-41-new** **CTAACGCAACCATCCTGCCAGGAATTGAAATTGGTGAAAAAGCAATCGTTGGTGCGGGGAGTGTTGTAACCAAAAATGTA**

**AB811624.1** **................................................................................**

**GU299792.1** **................................................................................**

**EU549863.1** **................................................................................**

3850 3860 3870 3880 3890 3900 3910 3920

....|....|....|....|....|....|....|....|....|....|....|....|....|....|....|....|

**U18-41-new** **CCGCCATGCGCAATAGTAGTAGGTAATCCAGCTCGATTTATTAAATGGGTAGAGGATAATGAATAAAATTGATTTTTTAG**

**AB811624.1** **................................................................................**

**GU299792.1** **................................................................................**

**EU549863.1** **................................................................................**

3930 3940 3950 3960 3970 3980 3990 4000

....|....|....|....|....|....|....|....|....|....|....|....|....|....|....|....|

**U18-41-new** **ATCTTTTTGCAATTAACCAGCGACAGCACAAAGAATTAGTCTCTGCGTTTAGTAGGGTGCTAGATTCTGGTTGGTATATC**

**AB811624.1** **................................................................................**

**GU299792.1** **................................................................................**

**EU549863.1** **................................................................................**

4010 4020 4030 4040 4050 4060 4070 4080

....|....|....|....|....|....|....|....|....|....|....|....|....|....|....|....|

**U18-41-new** **ATGGGCGAAGAACTTGAGCAGTTCGAGAAAGAGTTCGCAGAATACTGTGGAGTTAAGTATTGCATTGGTGTAGCAAATGG**

**AB811624.1** **................................................................................**

**GU299792.1** **................................................................................**

**EU549863.1** **................................................................................**

4090 4100 4110 4120 4130 4140 4150 4160

....|....|....|....|....|....|....|....|....|....|....|....|....|....|....|....|

**U18-41-new** **CCTTGATGCGTTGATACTAGTATTGAGGGCATGGAAAGAACTTGGCTATCTTGAAGACGGTGACGAGGTATTAGTACCGG**

**AB811624.1** **................................................................................**

**GU299792.1** **................................................................................**

**EU549863.1** **................................................................................**

4170 4180 4190 4200 4210 4220 4230 4240

....|....|....|....|....|....|....|....|....|....|....|....|....|....|....|....|

**U18-41-new** **CAAATACATATATTGCTTCTATTCTTGCTATAACAGAGAACAAACTTGTTCCTGTTCTTGTTGAACCAGATATAGAAACT**

**AB811624.1** **................................................................................**

**GU299792.1** **................................................................................**

**EU549863.1** **................................................................................**

4250 4260 4270 4280 4290 4300 4310 4320

....|....|....|....|....|....|....|....|....|....|....|....|....|....|....|....|

**U18-41-new** **TATAATATTAATCCTGCTTTAATTGAAAATTACATTACGGAAAAAACTAAAGCAATATTACCGGTTCGCTTATATGGTCT**

**AB811624.1** **................................................................................**

**GU299792.1** **...................................................................A............**

**EU549863.1** **...................................................................A............**

4330 4340 4350 4360 4370 4380 4390 4400

....|....|....|....|....|....|....|....|....|....|....|....|....|....|....|....|

**U18-41-new** **ATTGTGCAATATGCCAGAAATTAGTGCAATCGCCAGAAAATATAATCTGTTGATTCTTGAAGATTGTGCACAAGCACATG**

**AB811624.1** **................................................................................**

**GU299792.1** **................................................................................**

**EU549863.1** **................................................................................**

4410 4420 4430 4440 4450 4460 4470 4480

....|....|....|....|....|....|....|....|....|....|....|....|....|....|....|....|

**U18-41-new** **GTGCAATACGTGATGGTCGCAAAGCTGGAGCTTGGGGGGATGCTGCAGGATTTAGTTTTTATCCAGGAAAAAACCTTGGA**

**AB811624.1** **................................................................................**

**GU299792.1** **................................................................................**

**EU549863.1** **................................................................................**

4490 4500 4510 4520 4530 4540 4550 4560

....|....|....|....|....|....|....|....|....|....|....|....|....|....|....|....|

**U18-41-new** **GCTTTGGGGGATGCGGGAGCTGTTACTACAAATAATGCAGAATTATCCTCAACTATAAAAGCTTTGCGAAATTATGGGTC**

**AB811624.1** **................................................................................**

**GU299792.1** **................................................................................**

**EU549863.1** **................................................................................**

4570 4580 4590 4600 4610 4620 4630 4640

....|....|....|....|....|....|....|....|....|....|....|....|....|....|....|....|

**U18-41-new** **ACATAAGAAATATGAAAATATTTATCAGGGATTGAATAGTCGATTGGATGAACTGCAAGCAGCCTTATTGCGTGTAAAAA**

**AB811624.1** **................................................................................**

**GU299792.1** **................................................................................**

**EU549863.1** **................................................................................**

4650 4660 4670 4680 4690 4700 4710 4720

....|....|....|....|....|....|....|....|....|....|....|....|....|....|....|....|

**U18-41-new** **TCCATACATTACCGGAAGATACTGCGATTCGGCAAAGGATTGCTGAAAAATATATTCGTGAAATAAAAAACCCTGCGATT**

**AB811624.1** **................................................................................**

**GU299792.1** **................................................................................**

**EU549863.1** **................................................................................**

4730 4740 4750 4760 4770 4780 4790 4800

....|....|....|....|....|....|....|....|....|....|....|....|....|....|....|....|

**U18-41-new** **ACGTTACCAGTGTACGAAGGCCAAGGTGCGCACGTTTGGCATTTATTTGTAGTAAGAATCGCTAATCGTGAAAAATTCCA**

**AB811624.1** **................................................................................**

**GU299792.1** **................................................................................**

**EU549863.1** **................................................................................**

4810 4820 4830 4840 4850 4860 4870 4880

....|....|....|....|....|....|....|....|....|....|....|....|....|....|....|....|

**U18-41-new** **GTCATACTTATTAGAGAAGGGTATCAAAACCTTAATTCACTATCCATTACCACCCCATAAGCAGCAAGCATATCAAAATA**

**AB811624.1** **................................................................................**

**GU299792.1** **................................................................................**

**EU549863.1** **................................................................................**

4890 4900 4910 4920 4930 4940 4950 4960

....|....|....|....|....|....|....|....|....|....|....|....|....|....|....|....|

**U18-41-new** **TGTCTAGCCTTAGCCTTCCAATTACTGAGCAAATTCATGATGAAGTCATTTCTTTACCTATAAGTCCGGTAATGAGTGAA**

**AB811624.1** **................................................................................**

**GU299792.1** **................................................................................**

**EU549863.1** **................................................................................**

4970 4980 4990 5000 5010 5020 5030 5040

....|....|....|....|....|....|....|....|....|....|....|....|....|....|....|....|

**U18-41-new** **GATGATGTCAATTATGTAATCAAAATGGTCAATGATTACAAGTAATGAAAAAATTTCTTCAGGTAACTATATTATCCGCT**

**AB811624.1** **................................................................................**

**GU299792.1** **................................................................................**

**EU549863.1** **................................................................................**

5050 5060 5070 5080 5090 5100 5110 5120

....|....|....|....|....|....|....|....|....|....|....|....|....|....|....|....|

**U18-41-new** **ATCTATACATTCATTAAAATGATTGCGGGTTTTATCATCGGTAAGGTAGTAGCAATTTATACAGGGCCATCAGGGGTAGC**

**AB811624.1** **................................................................................**

**GU299792.1** **................................................................................**

**EU549863.1** **................................................................................**

5130 5140 5150 5160 5170 5180 5190 5200

....|....|....|....|....|....|....|....|....|....|....|....|....|....|....|....|

**U18-41-new** **AATGCTTGGCCAAGTGCAAAGTTTAATCACAATAGTTGCAGGTACTACCTCTGCACCTGTAAGCACTGGCCTTGTTCGAT**

**AB811624.1** **................................................................................**

**GU299792.1** **................................................................................**

**EU549863.1** **................................................................................**

5210 5220 5230 5240 5250 5260 5270 5280

....|....|....|....|....|....|....|....|....|....|....|....|....|....|....|....|

**U18-41-new** **ATACTGCGGAAAATTGGCAAGAAGGACAAGAAGCATGCGCGCCATGGTGGCGCGCATGCTTAAGGGTTACTCTGTTTTTA**

**AB811624.1** **................................................................................**

**GU299792.1** **................................................................................**

**EU549863.1** **................................................................................**

5290 5300 5310 5320 5330 5340 5350 5360

....|....|....|....|....|....|....|....|....|....|....|....|....|....|....|....|

**U18-41-new** **TTCTTGCTTATTATTCCCGTTGTTATTATATTGTCGAAAAATATTAGTGAGTTACTTTTTAGCGATGGACAATACACATG**

**AB811624.1** **................................................................................**

**GU299792.1** **................................................................................**

**EU549863.1** **................................................................................**

5370 5380 5390 5400 5410 5420 5430 5440

....|....|....|....|....|....|....|....|....|....|....|....|....|....|....|....|

**U18-41-new** **GTTAATCATTTTCGCATGTTGTATATTGCCATTCTCCATTATAAATACATTGATCGCTTCAGTTTTAAATGGTCAACAAT**

**AB811624.1** **................................................................................**

**GU299792.1** **................................................................................**

**EU549863.1** **................................................................................**

5450 5460 5470 5480 5490 5500 5510 5520

....|....|....|....|....|....|....|....|....|....|....|....|....|....|....|....|

**U18-41-new** **TTTATAAGCAATATATATTGGTTGGGATGTTTTCTGTATTCATTTCTACTATGTTTATGATTTTGTTGATTGTAGCTTAT**

**AB811624.1** **................................................................................**

**GU299792.1** **................................................................................**

**EU549863.1** **................................................................................**

5530 5540 5550 5560 5570 5580 5590 5600

....|....|....|....|....|....|....|....|....|....|....|....|....|....|....|....|

**U18-41-new** **AATCTTAAAGGTGCATTGATTGCCACAGCTATAAATAGTGCTATTGCTGGTCTTGTATTGGTTTTATTTTGTCTCAATAA**

**AB811624.1** **................................................................................**

**GU299792.1** **................................................................................**

**EU549863.1** **................................................................................**

5610 5620 5630 5640 5650 5660 5670 5680

....|....|....|....|....|....|....|....|....|....|....|....|....|....|....|....|

**U18-41-new** **ATCTTGGTTTAGATTTAAATATTGGTGGGGTAAAACGGATAAAGACAAAATTATAAAAATTATTCATTATACTCTGATGG**

**AB811624.1** **................................................................................**

**GU299792.1** **................................................................................**

**EU549863.1** **................................................................................**

5690 5700 5710 5720 5730 5740 5750 5760

....|....|....|....|....|....|....|....|....|....|....|....|....|....|....|....|

**U18-41-new** **CTCTGGTTTCTGTTATCTCCATGCCTACAGCATTGATGTGTATTAGAAAAATATTGATTGCTAAAACTGGTTGGGAGGAT**

**AB811624.1** **................................................................................**

**GU299792.1** **................................................................................**

**EU549863.1** **................................................................................**

5770 5780 5790 5800 5810 5820 5830 5840

....|....|....|....|....|....|....|....|....|....|....|....|....|....|....|....|

**U18-41-new** **GCAGGGCAATGGCAGGCCGTATGGAAGATATCTGAGGTTTATCTTGGTGTTGTGACAATTGCTTTGTCAACATATTTCTT**

**AB811624.1** **................................................................................**

**GU299792.1** **................................................................................**

**EU549863.1** **................................................................................**

5850 5860 5870 5880 5890 5900 5910 5920

....|....|....|....|....|....|....|....|....|....|....|....|....|....|....|....|

**U18-41-new** **ACCAAGATTGACAATTATAAAAACAAGTTTCCTTATAAAAAAAGAAGTAAATAGTACTATATTATACATAATATCTATTA**

**AB811624.1** **................................................................................**

**GU299792.1** **................................................................................**

**EU549863.1** **................................................................................**

5930 5940 5950 5960 5970 5980 5990 6000

....|....|....|....|....|....|....|....|....|....|....|....|....|....|....|....|

**U18-41-new** **CTTCATTCATGGCGTTGAGTATCTATTTATTCCGCGATTTGGTAATAACAGTTTTATTTACTGAACAGTTTCGCTCAGCT**

**AB811624.1** **................................................................................**

**GU299792.1** **................................................................................**

**EU549863.1** **................................................................................**

6010 6020 6030 6040 6050 6060 6070 6080

....|....|....|....|....|....|....|....|....|....|....|....|....|....|....|....|

**U18-41-new** **CGTGAATTATTTTTATTACAACTTATAGGGGATGTAATAAAAATTGCTGGGTTTCTTTATGCATACCCTCTTCAAAGTCA**

**AB811624.1** **................................................................................**

**GU299792.1** **................................................................................**

**EU549863.1** **................................................................................**

6090 6100 6110 6120 6130 6140 6150 6160

....|....|....|....|....|....|....|....|....|....|....|....|....|....|....|....|

**U18-41-new** **GGGGCATACTAAACTATTCATCAGTTCAGAAGTGATTTTTTCTATGCTCTTTATCATTACCACCTATATTTTTGTTGTAA**

**AB811624.1** **................................................................................**

**GU299792.1** **................................................................................**

**EU549863.1** **................................................................................**

6170 6180 6190 6200 6210 6220 6230 6240

....|....|....|....|....|....|....|....|....|....|....|....|....|....|....|....|

**U18-41-new** **ATTATGGAGTACATGGTGCTAACATAAGTTATGTCATTACATATAGTTTATATTTTGTGTTTGCATTTGTGTTTACTAAT**

**AB811624.1** **................................................................................**

**GU299792.1** **................................................................................**

**EU549863.1** **................................................................................**

6250 6260 6270 6280 6290 6300 6310 6320

....|....|....|....|....|....|....|....|....|....|....|....|....|....|....|....|

**U18-41-new** **TTTATTAATGTTAGAAGAAATAATTAAAAACAGAGGTTGAATTTTGAAAATAATTATACCTGTCTTAGGATTTGGCAGGG**

**AB811624.1** **................................................................................**

**GU299792.1** **................................................................................**

**EU549863.1** **................................................................................**

6330 6340 6350 6360 6370 6380 6390 6400

....|....|....|....|....|....|....|....|....|....|....|....|....|....|....|....|

**U18-41-new** **CTGGTGGTGAAAGAGTTCTTTCTAAGCTGGCAACTGAATTGATGAATTATGGACATGATGTAAGTTTTGTTGTTCCAGAT**

**AB811624.1** **................................................................................**

**GU299792.1** **.........................................G......................................**

**EU549863.1** **................................................................................**

6410 6420 6430 6440 6450 6460 6470 6480

....|....|....|....|....|....|....|....|....|....|....|....|....|....|....|....|

**U18-41-new** **AATAGAACTAATCCATATTATGCTACCACAGCAAAAATTGTCACGAGTAAATCTAGTCAAAACCGTGTAAAAATATTGAG**

**AB811624.1** **................................................................................**

**GU299792.1** **................................................................................**

**EU549863.1** **................................................................................**

6490 6500 6510 6520 6530 6540 6550 6560

....|....|....|....|....|....|....|....|....|....|....|....|....|....|....|....|

**U18-41-new** **AATCATTAAAAATTACTATAATCTGTGGCGTAAATGCATAGAGTTAAATCCTGATGCTGTAGTTGCTAGTTTTCATTTGA**

**AB811624.1** **................................................................................**

**GU299792.1** **................................................................................**

**EU549863.1** **................................................................................**

6570 6580 6590 6600 6610 6620 6630 6640

....|....|....|....|....|....|....|....|....|....|....|....|....|....|....|....|

**U18-41-new** **CTGCCTATCTTGTCGCATTATTACCAATCACCCGTCGTAAGAAATATTATTATATTCAGGCGTATGAAGTTAATTTTTTT**

**AB811624.1** **................................................................................**

**GU299792.1** **................................................................................**

**EU549863.1** **................................................................................**

6650 6660 6670 6680 6690 6700 6710 6720

....|....|....|....|....|....|....|....|....|....|....|....|....|....|....|....|

**U18-41-new** **GATAATATAATATGGAAATTAATAGCGGGTTTAACATATTATTTACCGCTTAAAAAAATACTAAATAGTCCTAATTTGCT**

**AB811624.1** **................................................................................**

**GU299792.1** **................................................................................**

**EU549863.1** **................................................................................**

6730 6740 6750 6760 6770 6780 6790 6800

....|....|....|....|....|....|....|....|....|....|....|....|....|....|....|....|

**U18-41-new** **TCCTCATAAACATGATGATTTTATAGGAGTAGTTCCTGCAGGAGTAGATTTAAACGTTTTCTATCCGAAACCATCAAATA**

**AB811624.1** **................................................................................**

**GU299792.1** **................................................................................**

**EU549863.1** **................................................................................**

6810 6820 6830 6840 6850 6860 6870 6880

....|....|....|....|....|....|....|....|....|....|....|....|....|....|....|....|

**U18-41-new** **GGTTATTAAATGGTCACACATCAATAGGGATTATTGGTAGAAAAGAGAAGCACAAAGGAACTAGCGAAATTATTTCAGTA**

**AB811624.1** **................................................................................**

**GU299792.1** **................................................................................**

**EU549863.1** **................................................................................**

6890 6900 6910 6920 6930 6940 6950 6960

....|....|....|....|....|....|....|....|....|....|....|....|....|....|....|....|

**U18-41-new** **TTGTGTTCACTGGAAAATAAAGCTGGAATTATAATCAATATTGCGATCTATCTTGAAGAAGTTGATAAGCAGCGTTTAAT**

**AB811624.1** **................................................................................**

**GU299792.1** **................................................................................**

**EU549863.1** **................................................................................**

6970 6980 6990 7000 7010 7020 7030 7040

....|....|....|....|....|....|....|....|....|....|....|....|....|....|....|....|

**U18-41-new** **CGCTGCCGGGTTTCAGGTTAATTTTTTTCCGATTACTTCTGATTTAGAATTGGCATCCTTTTATCGAAGCAATGACATCA**

**AB811624.1** **................................................................................**

**GU299792.1** **................................................................................**

**EU549863.1** **................................................................................**

7050 7060 7070 7080 7090 7100 7110 7120

....|....|....|....|....|....|....|....|....|....|....|....|....|....|....|....|

**U18-41-new** **TGATTGCTGTTGGGTTAATTGAAGATGGCGCTTTCCATTATCCTTGTGCTGAATCAATGGCTTGTGGTTGTCTTGTTATT**

**AB811624.1** **................................................................................**

**GU299792.1** **................................................................................**

**EU549863.1** **................................................................................**

7130 7140 7150 7160 7170 7180 7190 7200

....|....|....|....|....|....|....|....|....|....|....|....|....|....|....|....|

**U18-41-new** **TCAAATTATGCGCCACTTACTGAAACTAACAGTGTACTTAAATTAGTCAAGTTTGATGCTTGCAAACTTGGTGAAGCAAT**

**AB811624.1** **................................................................................**

**GU299792.1** **................................................................................**

**EU549863.1** **................................................................................**

7210 7220 7230 7240 7250 7260 7270 7280

....|....|....|....|....|....|....|....|....|....|....|....|....|....|....|....|

**U18-41-new** **TAATCTTTGTCTCAATCTTGACCTAGAAGAAAAAAGCAAAGAAATCCAATCTAATATTTCTGTGTTGAATAAATATGACT**

**AB811624.1** **................................................................................**

**GU299792.1** **................................................................................**

**EU549863.1** **................................................................................**

7290 7300 7310 7320 7330 7340 7350 7360

....|....|....|....|....|....|....|....|....|....|....|....|....|....|....|....|

**U18-41-new** **GGAAAATTGTTGGTGAAACTTTCAATAGTTTATTGTTAGATGCAAATAAATAGTATACGTTGATGGGGAAAATATGAATA**

**AB811624.1** **................................................................................**

**GU299792.1** **................................................................................**

**EU549863.1** **................................................................................**

7370 7380 7390 7400 7410 7420 7430 7440

....|....|....|....|....|....|....|....|....|....|....|....|....|....|....|....|

**U18-41-new** **TTGTTAAAACTGATATTCCAGATCTGATCGTTCTTGAACCAAAAGTGTTTAGTGATGAACGCGGCTTTTTTATGGAGAGT**

**AB811624.1** **................................................................................**

**GU299792.1** **................................................................................**

**EU549863.1** **................................................................................**

7450 7460 7470 7480 7490 7500 7510 7520

....|....|....|....|....|....|....|....|....|....|....|....|....|....|....|....|

**U18-41-new** **TATAATCAGATTGAATTTGAGAAGGCAATAGGAAGGCACGTAAATTTTGTTCAGGATAATCATTCAAAATCTAGTAAAGG**

**AB811624.1** **................................................................................**

**GU299792.1** **................................................................................**

**EU549863.1** **................................................................................**

7530 7540 7550 7560 7570 7580 7590 7600

....|....|....|....|....|....|....|....|....|....|....|....|....|....|....|....|

**U18-41-new** **CGTACTACGTGGGTTGCATTATCAATTAGCACCGTATGCACAGGCTAAATTAGTTCGATGTGTTGTAGGTCAGGTATTTG**

**AB811624.1** **................................................................................**

**GU299792.1** **................................................................................**

**EU549863.1** **................................................................................**

7610 7620 7630 7640 7650 7660 7670 7680

....|....|....|....|....|....|....|....|....|....|....|....|....|....|....|....|

**U18-41-new** **ATGTTGCTGTTGATCTTAGAAAAAATTCACCAACGTTCAAAAAATGGTTTGGAATAACCCTTTCCGCAGAAAATAAACGA**

**AB811624.1** **................................................................................**

**GU299792.1** **................................................................................**

**EU549863.1** **................................................................................**

7690 7700 7710 7720 7730 7740 7750 7760

....|....|....|....|....|....|....|....|....|....|....|....|....|....|....|....|

**U18-41-new** **CAATTATGGATACCCGAAGGATTTGCTCATGGTTTCTTGGTGACCAGTGATGAAGCTGAGTTCATTTATAAGACAACTAA**

**AB811624.1** **................................................................................**

**GU299792.1** **................................................................................**

**EU549863.1** **................................................................................**

7770 7780 7790 7800 7810 7820 7830 7840

....|....|....|....|....|....|....|....|....|....|....|....|....|....|....|....|

**U18-41-new** **CTACTATGCTCCTGGTCATCAGCAAGCAATTATTTACAATGATCCTATTTTAAACATCGATTGGCCTTTCTGCAGTAGTG**

**AB811624.1** **................................................................................**

**GU299792.1** **................................................................................**

**EU549863.1** **................................................................................**

7850 7860 7870 7880 7890 7900 7910 7920

....|....|....|....|....|....|....|....|....|....|....|....|....|....|....|....|

**U18-41-new** **CTCTGTCATTATCACAAAAAGATCAAGAAGCAAAATTATTTTCAGAATTATTGGACAGTGAACTGTTCTAATAAAGTGTG**

**AB811624.1** **................................................................................**

**GU299792.1** **................................................................................**

**EU549863.1** **................................................................................**

7930 7940 7950 7960 7970 7980 7990 8000

....|....|....|....|....|....|....|....|....|....|....|....|....|....|....|....|

**U18-41-new** **CCACCTTATCCGTCTGAAGGATAGGTGGTTGCTTATATTTTTTT-GAGTATGTTTGTATAATGACAGAAAATAGTCCGAA**

**AB811624.1** **............................................-...................................**

**GU299792.1** **............................................T...................................**

**EU549863.1** **............................................-...................................**

8010 8020 8030 8040 8050 8060 8070 8080

....|....|....|....|....|....|....|....|....|....|....|....|....|....|....|....|

**U18-41-new** **ATATAAACACGATAAAAGCTTAATAAGTTTTATCTACTTATTTTTTATATTTACACTTATTGTAGGCTTTATTATCGCAA**

**AB811624.1** **................................................................................**

**GU299792.1** **................................................................................**

**EU549863.1** **................................................................................**

8090 8100 8110 8120 8130 8140 8150 8160

....|....|....|....|....|....|....|....|....|....|....|....|....|....|....|....|

**U18-41-new** **TTACCCAGTTTTTGGGGCGAAGTAGAGACTATGATAATTATATACAGATCTTTTCTGGTAAAGAAGGGGAGGGGGTTCTT**

**AB811624.1** **................................................................................**

**GU299792.1** **A...............................................................................**

**EU549863.1** **A...............................................................................**

8170 8180 8190 8200 8210 8220 8230 8240

....|....|....|....|....|....|....|....|....|....|....|....|....|....|....|....|

**U18-41-new** **GAATTATTTTATCGCGGATTGATGTTAATAACGACCAGCTATGAAACTATCATTTTTATAATTTTAACATGTTCTTTTTT**

**AB811624.1** **................................................................................**

**GU299792.1** **................................................................................**

**EU549863.1** **................................................................................**

8250 8260 8270 8280 8290 8300 8310 8320

....|....|....|....|....|....|....|....|....|....|....|....|....|....|....|....|

**U18-41-new** **TATAAAGGCAAGGTTTCTCGCTAACTATTCGCGTAATTTTTCAGGCTTGACCTTATTCTTTATTTATTATGCAAGCGTTG**

**AB811624.1** **................................................................................**

**GU299792.1** **................................................................................**

**EU549863.1** **................................................................................**

8330 8340 8350 8360 8370 8380 8390 8400

....|....|....|....|....|....|....|....|....|....|....|....|....|....|....|....|

**U18-41-new** **CACTTTGGGTTTTAGATTATACTCAATTCAGAAATGGTCTATGTATTTCCATTTTAATGTTTTCCGTATACTATTTATTT**

**AB811624.1** **................................................................................**

**GU299792.1** **................................................................................**

**EU549863.1** **................................................................................**

8410 8420 8430 8440 8450 8460 8470 8480

....|....|....|....|....|....|....|....|....|....|....|....|....|....|....|....|

**U18-41-new** **ATAAATAAACCGACTTATTTTTATTTCTCGGTATTATGTGCAATTGCAACTCATTGGTCTGCTTTGCCTTTTTTGCTTTT**

**AB811624.1** **................................................................................**

**GU299792.1** **................................................................................**

**EU549863.1** **................................................................................**

8490 8500 8510 8520 8530 8540 8550 8560

....|....|....|....|....|....|....|....|....|....|....|....|....|....|....|....|

**U18-41-new** **ATATCCTTTTGTCTATTCAACAAAAATAAGACGCCTTGGTTATTTTTGTTTCAGTATTCTTGTTTTGATTGCGATCTCAG**

**AB811624.1** **................................................................................**

**GU299792.1** **................................................................................**

**EU549863.1** **................................................................................**

8570 8580 8590 8600 8610 8620 8630 8640

....|....|....|....|....|....|....|....|....|....|....|....|....|....|....|....|

**U18-41-new** **GAGAAGGAAAAGAGATCATATCTTTTATAAGAAATTTTGGAGTGGGACAAAAAATAGGAAATGAAGCTGGTGTAAATTTA**

**AB811624.1** **................................................................................**

**GU299792.1** **................................................................................**

**EU549863.1** **................................................................................**

8650 8660 8670 8680 8690 8700 8710 8720

....|....|....|....|....|....|....|....|....|....|....|....|....|....|....|....|

**U18-41-new** **ATAAATTCATTATCCCTTACCGCTATTTCCTGGTTTATTATTAGTTACATATCAAGCATTGGAAATGAAAGGAGAAATTT**

**AB811624.1** **................................................................................**

**GU299792.1** **................................................................................**

**EU549863.1** **................................................................................**

8730 8740 8750 8760 8770 8780 8790 8800

....|....|....|....|....|....|....|....|....|....|....|....|....|....|....|....|

**U18-41-new** **AAGGCTTTTCTTTTGTTATGGTGTCATGCAATACGTGACTTTTAGCCTTTTCTCTCTACCTGTTATGGCTTTCCGTATTT**

**AB811624.1** **................................................................................**

**GU299792.1** **................................................................................**

**EU549863.1** **................................................................................**

8810 8820 8830 8840 8850 8860 8870 8880

....|....|....|....|....|....|....|....|....|....|....|....|....|....|....|....|

**U18-41-new** **TGGAAATGTATTTTTTCCTTATGCTAACCATTGGGGTGTTTATTAAGCAAAAAAAGAATTATTATTTTGTTTTTTGCAAA**

**AB811624.1** **................................................................................**

**GU299792.1** **................................................................................**

**EU549863.1** **................................................................................**

8890 8900 8910 8920 8930 8940 8950 8960

....|....|....|....|....|....|....|....|....|....|....|....|....|....|....|....|

**U18-41-new** **GTGTTAATTTTATTGTATCTAACATACTATTATCATATGGTCTTTGGAGTGATTAATGTGTAAGGCTAAGGTGTTGGCTA**

**AB811624.1** **................................................................................**

**GU299792.1** **................................................................................**

**EU549863.1** **................................................................................**

8970 8980 8990 9000 9010 9020 9030 9040

....|....|....|....|....|....|....|....|....|....|....|....|....|....|....|....|

**U18-41-new** **TAATTGTTACTTACAACCCGGAAATTATTCGATTGACGGAATGTATTAACTCTTTAGCCCCACAAGTTGAGAGAATAATT**

**AB811624.1** **................................................................................**

**GU299792.1** **................................................................................**

**EU549863.1** **................................................................................**

9050 9060 9070 9080 9090 9100 9110 9120

....|....|....|....|....|....|....|....|....|....|....|....|....|....|....|....|

**U18-41-new** **CTTGTAGATAATGGCTCAAATAATAGTGATTTGATAAAAAATATCAGTATTAATAACCTTGAAATTATTTTACTTTCGGA**

**AB811624.1** **................................................................................**

**GU299792.1** **................................................................................**

**EU549863.1** **................................................................................**

9130 9140 9150 9160 9170 9180 9190 9200

....|....|....|....|....|....|....|....|....|....|....|....|....|....|....|....|

**U18-41-new** **AAACAAAGGCATTGCATTTGCTCAGAACCATGGTGTTAAGAAGGGCCTGGAAGCAAAAGAGTTTGACTATTTATTTTTCT**

**AB811624.1** **................................................................................**

**GU299792.1** **................................................................................**

**EU549863.1** **................................................................................**

9210 9220 9230 9240 9250 9260 9270 9280

....|....|....|....|....|....|....|....|....|....|....|....|....|....|....|....|

**U18-41-new** **CAGATCAGGATACTTGCTTTCCTAGCGATGTTATTGAAAAACTTAAGAGTACATTTACGAAAAATAATAAAAAAGGTAAA**

**AB811624.1** **................................................................................**

**GU299792.1** **................................................................................**

**EU549863.1** **................................................................................**

9290 9300 9310 9320 9330 9340 9350 9360

....|....|....|....|....|....|....|....|....|....|....|....|....|....|....|....|

**U18-41-new** **AATGTTGCTTGTGCTTCTCCTTTTTTTAAAGACCATCGTTCAAATTATATGCATCCGTCAGTCAGCCTAAATATTTTTAC**

**AB811624.1** **................................................................................**

**GU299792.1** **................................................................................**

**EU549863.1** **................................................................................**

9370 9380 9390 9400 9410 9420 9430 9440

....|....|....|....|....|....|....|....|....|....|....|....|....|....|....|....|

**U18-41-new** **GAGTACAAAAGTTATATGTAGTGAAGTAGACGATGATCTTTATCCCTCGCATGTTATTGCTTCTGGGATGTTAATGTCTC**

**AB811624.1** **................................................................................**

**GU299792.1** **..............................................C.................................**

**EU549863.1** **................................................................................**

9450 9460 9470 9480 9490 9500 9510 9520

....|....|....|....|....|....|....|....|....|....|....|....|....|....|....|....|

**U18-41-new** **GTGAAGCATGGCGCGTCGTCGGACCATTTTGTGAAAAACTCTTTATAGACTGGGTTGATACAGAATGGTGTTGGCGTGCA**

**AB811624.1** **................................................................................**

**GU299792.1** **................................................................................**

**EU549863.1** **................................................................................**

9530 9540 9550 9560 9570 9580 9590 9600

....|....|....|....|....|....|....|....|....|....|....|....|....|....|....|....|

**U18-41-new** **TTAGCTAATAATATGATTATTGTTCAGACACCATCAGTCATCATTTCTCATGAACTTGGGTATGGGCAGAAAATTTTTGC**

**AB811624.1** **................................................................................**

**GU299792.1** **................................................................................**

**EU549863.1** **................................................................................**

9610 9620 9630 9640 9650 9660 9670 9680

....|....|....|....|....|....|....|....|....|....|....|....|....|....|....|....|

**U18-41-new** **TGGTCGATCTGTTACAATACATAATTCTTTCAGAAATTTTTATAAAATACGCAATGCAATATACTTAATGCTGCATTCAA**

**AB811624.1** **................................................................................**

**GU299792.1** **................................................................................**

**EU549863.1** **................................................................................**

9690 9700 9710 9720 9730 9740 9750 9760

....|....|....|....|....|....|....|....|....|....|....|....|....|....|....|....|

**U18-41-new** **ATTATAGCTTCAAGTATCGTTATCATGCTTTTTTTCATGCGACAAAGAATGTTGTATTTGAAATTTTATATTCGAAAGAA**

**AB811624.1** **................................................................................**

**GU299792.1** **................................................................................**

**EU549863.1** **................................................................................**

9770 9780 9790 9800 9810 9820 9830 9840

....|....|....|....|....|....|....|....|....|....|....|....|....|....|....|....|

**U18-41-new** **AAATTAAATTCACTGAAGGTTTGTTTTAAAGCTGTACGTGATGGTATGTTCAATAATTTTTAATACGAAAATAGTTAGGC**

**AB811624.1** **................................................................................**

**GU299792.1** **................................................................................**

**EU549863.1** **................................................................................**

9850 9860 9870 9880 9890 9900 9910 9920

....|....|....|....|....|....|....|....|....|....|....|....|....|....|....|....|

**U18-41-new** **TCAAGGTGTTTAAATGGAAGAAAATAATATGAAGACGGTCGCTGTAGTTGGCACAGTGGGTGTTCCTGCTTGTTATGGTG**

**AB811624.1** **................................................................................**

**GU299792.1** **................................................................................**

**EU549863.1** **................................................................................**

9930 9940 9950 9960 9970 9980 9990 10000

....|....|....|....|....|....|....|....|....|....|....|....|....|....|....|....|

**U18-41-new** **GGTTCGAATCACTTGTTCAGAATCTAATTGATTATCAATCTGATGGTATACAATATCAGATATTTTGCTCTTCAAAAAAA**

**AB811624.1** **................................................................................**

**GU299792.1** **................................................................................**

**EU549863.1** **................................................................................**

10010 10020 10030 10040 10050 10060 10070 10080

....|....|....|....|....|....|....|....|....|....|....|....|....|....|....|....|

**U18-41-new** **TATGATAAAAAATTTAAAAATTATAAAAATGCAGAATTAATCTATTTGCCGATAAATGCCAATGGCGTCTCTAGCATAAT**

**AB811624.1** **................................................................................**

**GU299792.1** **................................................................................**

**EU549863.1** **................................................................................**

10090 10100 10110 10120 10130 10140 10150 10160

....|....|....|....|....|....|....|....|....|....|....|....|....|....|....|....|

**U18-41-new** **TTATGATATTATGTGTTTAATTATTTGTTTATTCAAAAGGCCAGATGTTGTTTTAATATTGGGGGTGTCTGGTTGTTTAT**

**AB811624.1** **................................................................................**

**GU299792.1** **................................................................................**

**EU549863.1** **................................................................................**

10170 10180 10190 10200 10210 10220 10230 10240

....|....|....|....|....|....|....|....|....|....|....|....|....|....|....|....|

**U18-41-new** **TTCTACCAATTTATAAACTATTTTCAAAATCAAAGATTATTGTCAATATTGATGGGCTTGAATGGCGTAGAAATAAATGG**

**AB811624.1** **................................................................................**

**GU299792.1** **................................................................................**

**EU549863.1** **................................................................................**

10250 10260 10270 10280 10290 10300 10310 10320

....|....|....|....|....|....|....|....|....|....|....|....|....|....|....|....|

**U18-41-new** **GGAACGTTTGCTAAGAAATTTCTTAAAATATCTGAGGCGATATCTATTAGAATAGCTGATATTATCATTTCAGATAATCA**

**AB811624.1** **................................................................................**

**GU299792.1** **................................................................................**

**EU549863.1** **................................................................................**

10330 10340 10350 10360 10370 10380 10390 10400

....|....|....|....|....|....|....|....|....|....|....|....|....|....|....|....|

**U18-41-new** **AGCAATAGCTGATTATGTGGAAAATAAGTACAAGAAAAAAAGTGTAGTTATAGCTTATGGCGGAGATCATGCCACTAATC**

**AB811624.1** **................................................................................**

**GU299792.1** **................................................................................**

**EU549863.1** **................................................................................**

10410 10420 10430 10440 10450 10460 10470 10480

....|....|....|....|....|....|....|....|....|....|....|....|....|....|....|....|

**U18-41-new** **TTAGTACACCGATAGACAATGATCAAAAAAAAGAAGGTTATTATTTGGGGCTTTGTAGGATAGAGCCTGAGAATAATATA**

**AB811624.1** **................................................................................**

**GU299792.1** **................................................................................**

**EU549863.1** **................................................................................**

10490 10500 10510 10520 10530 10540 10550 10560

....|....|....|....|....|....|....|....|....|....|....|....|....|....|....|....|

**U18-41-new** **GAAATGATTCTGAATGCCTTCATTAATACAGATAAAAAAATTAAATTTATGGGTAATTGGGATAACAGCGAGTATGGACG**

**AB811624.1** **................................................................................**

**GU299792.1** **................................................................................**

**EU549863.1** **................................................................................**

10570 10580 10590 10600 10610 10620 10630 10640

....|....|....|....|....|....|....|....|....|....|....|....|....|....|....|....|

**U18-41-new** **CCAGCTAAAAAAATATTATTCAAACTATCCAAATATCACCCTACTAGAACCTAACTATAATATTGAAGAGCTTTATAAAC**

**AB811624.1** **................................................................................**

**GU299792.1** **................................................................................**

**EU549863.1** **................................................................................**

10650 10660 10670 10680 10690 10700 10710 10720

....|....|....|....|....|....|....|....|....|....|....|....|....|....|....|....|

**U18-41-new** **TAAGAAAAAATTGTCTTGCATACATTCATGGACACTCGGCTGGTGGAACAAACCCTTCTTTAGTTGAAGCGATGCATTTT**

**AB811624.1** **................................................................................**

**GU299792.1** **................................................................................**

**EU549863.1** **................................................................................**

10730 10740 10750 10760 10770 10780 10790 10800

....|....|....|....|....|....|....|....|....|....|....|....|....|....|....|....|

**U18-41-new** **AATATTCCTATTTTTGCTTTCGATTGTGACTTTAATCGTTACACAACTAACAATTTAGCTCATTACTTTAATGATTCTGA**

**AB811624.1** **................................................................................**

**GU299792.1** **................................................................................**

**EU549863.1** **................................................................................**

10810 10820 10830 10840 10850 10860 10870 10880

....|....|....|....|....|....|....|....|....|....|....|....|....|....|....|....|

**U18-41-new** **ACAACTTAGCTTATTAGCAGAAAGTTTGTCTTTTGGAAATCTTAAATGTCGAGTATTAGATTTAAAAAATTATGCTGAAG**

**AB811624.1** **................................................................................**

**GU299792.1** **................................................................................**

**EU549863.1** **................................................................................**

10890 10900 10910 10920 10930 10940 10950 10960

....|....|....|....|....|....|....|....|....|....|....|....|....|....|....|....|

**U18-41-new** **ATATGTATAACTGGAGGCATATAGCTGCTATGTATGAATCTATTTATTAAACGCATTAACAATAATATAATTGACCTTAT**

**AB811624.1** **................................................................................**

**GU299792.1** **................................................................................**

**EU549863.1** **................................................................................**

10970 10980 10990 11000 11010 11020 11030 11040

....|....|....|....|....|....|....|....|....|....|....|....|....|....|....|....|

**U18-41-new** **ATAGCAGGGAAAGATCACGTAACGCTGCTTTTTTGTACTAAATAATTCGCATTTTATGTTTAAAAATTGAGATATCCCTT**

**AB811624.1** **................................................................................**

**GU299792.1** **................................................................................**

**EU549863.1** **................................................................................**

11050 11060 11070 11080 11090 11100 11110 11120

....|....|....|....|....|....|....|....|....|....|....|....|....|....|....|....|

**U18-41-new** **ATTACCTAAAGCTGTTTTTTATTGCTTATAC-ATGATCAAATACTCCTTACATAATTAAGGAGAACAAAATGGAACTTAA**

**AB811624.1** **...............................-................................................**

**GU299792.1** **...............................-................................................**

**EU549863.1** **...............................C................................................**

11130 11140 11150 11160 11170 11180 11190 11200

....|....|....|....|....|....|....|....|....|....|....|....|....|....|....|....|

**U18-41-new** **AAAATTGATGGAACATATTTCTATTATCCCCGATTACAGACAAGCCTGGAAAGTGGAACATAAATTATCGGATATTCTAC**

**AB811624.1** **................................................................................**

**GU299792.1** **................................................................................**

**EU549863.1** **................................................................................**

11210 11220 11230 11240 11250 11260 11270 11280

....|....|....|....|....|....|....|....|....|....|....|....|....|....|....|....|

**U18-41-new** **TGTTGACTATTTGTGCCGTTATTTCGGGTGCAGAAGGCTGGGAAGATATAGAGGATTTTGGGGAAACACATCTCGATTTT**

**AB811624.1** **................................................................................**

**GU299792.1** **................................................................................**

**EU549863.1** **................................................................................**

11290 11300 11310 11320 11330 11340 11350 11360

....|....|....|....|....|....|....|....|....|....|....|....|....|....|....|....|

**U18-41-new** **TTGAAGCAATATGGTGATTTTGATAATGGTATTCCTGTTCACGATACTATTGCCAGAGTTGTATCCTGTATCAGTCCTGC**

**AB811624.1** **................................................................................**

**GU299792.1** **................................................................................**

**EU549863.1** **........................--------------------------------------------------------**

11370 11380 11390 11400 11410 11420 11430 11440

....|....|....|....|....|....|....|....|....|....|....|....|....|....|....|....|

**U18-41-new** **AAAGTTTCACGAGTGCTTTATTAACTGGATGCGTGACTGCCATTCTTCAGATGATAAAGACGTCATCGCAATTGATGGAA**

**AB811624.1** **................................................................................**

**GU299792.1** **................................................................................**

**EU549863.1** **--------------------------------------------------------------------------------**

11450 11460 11470 11480 11490 11500 11510 11520

....|....|....|....|....|....|....|....|....|....|....|....|....|....|....|....|

**U18-41-new** **AAACACTCCGGCATTCTTATGACAAGAGTCGCCGCAAGGGAGCGATTCATGTCATTAGTGCGTTCTCAACAATGCACAGT**

**AB811624.1** **................................................................................**

**GU299792.1** **................................................................................**

**EU549863.1** **--------------------------------------------------------------------------------**

11530 11540 11550 11560 11570 11580 11590 11600

....|....|....|....|....|....|....|....|....|....|....|....|....|....|....|....|

**U18-41-new** **CTGGTCATCGGACAGATCAGGACGGATGAGAAATCCAATGAAATTACAGCTATCCCTGAACTTCTTAACATGCTGGATAT**

**AB811624.1** **................................................................................**

**GU299792.1** **................................................................................**

**EU549863.1** **--------------------------------------------------------------------------------**

11610 11620 11630 11640 11650 11660 11670 11680

....|....|....|....|....|....|....|....|....|....|....|....|....|....|....|....|

**U18-41-new** **TAAAGGAAAAATCATCACAACTGATGCGATGGGGTGCCAGAAAGATATTGCAGAGAAGATATAAAAACAGGGCGGTGATT**

**AB811624.1** **................................................................................**

**GU299792.1** **................................................................................**

**EU549863.1** **--------------------------------------------------------------------------------**

11690 11700 11710 11720 11730 11740 11750 11760

....|....|....|....|....|....|....|....|....|....|....|....|....|....|....|....|

**U18-41-new** **ATTTATTCGCTGTAAAAGGAAATCAGGGGCGGCTTAATAAAGCCTTTGAGGAAAAATTTCCACTGAAAGAATTAAATAAT**

**AB811624.1** **................................................................................**

**GU299792.1** **................................................................................**

**EU549863.1** **--------------------------------------------------------------------------------**

11770 11780 11790 11800 11810 11820 11830 11840

....|....|....|....|....|....|....|....|....|....|....|....|....|....|....|....|

**U18-41-new** **CCAGAACATGACAGTTACGCCATTAGTGAAAAGAGTCACGGCAGAGAAGAAATCCGTCTTCATATTGTTTGCGATGTCCC**

**AB811624.1** **................................................................................**

**GU299792.1** **................................................................................**

**EU549863.1** **--------------------------------------------------------------------------------**

11850 11860 11870 11880 11890 11900 11910 11920

....|....|....|....|....|....|....|....|....|....|....|....|....|....|....|....|

**U18-41-new** **TGATGAACTTATTGATTTCACATTTGAATGGAAAGGACTGAAGAAATTATACGTTGCAGTCTCCTTTCGGTCCATAATAG**

**AB811624.1** **................................................................................**

**GU299792.1** **................................................................................**

**EU549863.1** **--------------------------------------------------------------------------------**

11930 11940 11950 11960 11970 11980 11990 12000

....|....|....|....|....|....|....|....|....|....|....|....|....|....|....|....|

**U18-41-new** **CAGAACAAAAGAAAGAGCCAGAAATGACGGTCAGATATTATATCAGTTCTGCTGATTTAACCGCAGAGAAGTTCGCCACA**

**AB811624.1** **................................................................................**

**GU299792.1** **................................................................................**

**EU549863.1** **--------------------------------------------------------------------------------**

12010 12020 12030 12040 12050 12060 12070 12080

....|....|....|....|....|....|....|....|....|....|....|....|....|....|....|....|

**U18-41-new** **GCAATCCGAAACCACTGGCACGTGGAGAATAAGCTGCACTGGCGTCTGGACGTGGTAATGAATGAAGACGACTGCAAAAT**

**AB811624.1** **................................................................................**

**GU299792.1** **................................................................................**

**EU549863.1** **--------------------------------------------------------------------------------**

12090 12100 12110 12120 12130 12140 12150 12160

....|....|....|....|....|....|....|....|....|....|....|....|....|....|....|....|

**U18-41-new** **AAGAAGAGGAAACGCCGCAGAATTATTTTCAGGGATACGGCACATCGCTATTAATATTTTAACGAATGATAAGGTATTCA**

**AB811624.1** **................................................................................**

**GU299792.1** **................................................................................**

**EU549863.1** **--------------------------------------------------------------------------------**

12170 12180 12190 12200 12210 12220 12230 12240

....|....|....|....|....|....|....|....|....|....|....|....|....|....|....|....|

**U18-41-new** **AGGCAGGGTTAAGACGTAAGATGCGAAAAGCAGCCATGGACAGAAACTATCTCGCGTCAGTCCTTGCGGGGAGCGGGCTT**

**AB811624.1** **................................................................................**

**GU299792.1** **................................................................................**

**EU549863.1** **--------------------------------------------------------------------------------**

12250 12260 12270 12280 12290 12300 12310 12320

....|....|....|....|....|....|....|....|....|....|....|....|....|....|....|....|

**U18-41-new** **TCGTAATCTTGCCCTGCCTTATATAGCTATCAGAGTAAAAAATGTAACGCTTATTATTTTGTTAAATAATACGCATTATA**

**AB811624.1** **................................................................................**

**GU299792.1** **................................................................................**

**EU549863.1** **--------------------------------------------------------------------------------**

12330 12340 12350 12360 12370 12380 12390 12400

....|....|....|....|....|....|....|....|....|....|....|....|....|....|....|....|

**U18-41-new** **TATTCAATAATTAAAATGCTCTTAATTAAATAAAACTATTTAATTAATAATATAAAATTTCATTATGTTACTTAAGAGAT**

**AB811624.1** **................................................................................**

**GU299792.1** **................................................................................**

**EU549863.1** **--------------------------------------------------------------------------------**

12410 12420 12430 12440 12450 12460 12470 12480

....|....|....|....|....|....|....|....|....|....|....|....|....|....|....|....|

**U18-41-new** **GAAAATTATAACTTAAAAAAGTGATGGAACATATTTCTATTATCCCTGATTACAGACAAGCCTGGAAAGTGGAACATAAA**

**AB811624.1** **................................................................................**

**GU299792.1** **................................................................................**

**EU549863.1** **--------------------------------------------------------------------------------**

12490 12500 12510 12520 12530 12540 12550 12560

....|....|....|....|....|....|....|....|....|....|....|....|....|....|....|....|

**U18-41-new** **TTGTCGGATATTCTACTGTTGACTATTTGTGCCGTTATTTTTGGTGCAGAAGGTTGGGAAATATAGAGGATTTTGGGGAA**

**AB811624.1** **................................................................................**

**GU299792.1** **................................................................................**

**EU549863.1** **--------------------------------------------------------------------------------**

12570 12580 12590 12600 12610 12620 12630 12640

....|....|....|....|....|....|....|....|....|....|....|....|....|....|....|....|

**U18-41-new** **ACACATCTCGTTTTTTTGAAGCAATATGGTGATTTTGAACATGGTATTCCTGCTCACGATACCATTGCCAGAGTTGTATC**

**AB811624.1** **................................................................................**

**GU299792.1** **................................................................................**

**EU549863.1** **---------------------------------------.........................................**

12650 12660 12670 12680 12690 12700 12710 12720

....|....|....|....|....|....|....|....|....|....|....|....|....|....|....|....|

**U18-41-new** **CTGTATCAGTCCTGCAAAATTTCACGAGTGCTTTATTAACTGGATGCGTGATTGTTATTCTTCATATGATAAAGACGTCA**

**AB811624.1** **................................................................................**

**GU299792.1** **................................................................................**

**EU549863.1** **................................................................................**

12730 12740 12750 12760 12770 12780 12790 12800

....|....|....|....|....|....|....|....|....|....|....|....|....|....|....|....|

**U18-41-new** **TCGCAATTGATGGAAAAACACTCCGGCATTCTTATGACAAGAGTCGCCGCAAGGGAGCGATTCATGTCATTAGTGCGTTC**

**AB811624.1** **................................................................................**

**GU299792.1** **................................................................................**

**EU549863.1** **................................................................................**

12810 12820 12830 12840 12850 12860 12870 12880

....|....|....|....|....|....|....|....|....|....|....|....|....|....|....|....|

**U18-41-new** **TCAACAATGCACAGTCTGGTCATCGGACAGATCAAGACGGATGAGAAATTCAATGAGGTCACAGCTATCCCAGAACTTCT**

**AB811624.1** **................................................................................**

**GU299792.1** **................................................................................**

**EU549863.1** **................................................................................**

12890 12900 12910 12920 12930 12940 12950 12960

....|....|....|....|....|....|....|....|....|....|....|....|....|....|....|....|

**U18-41-new** **TAACATATGGATATTAAAGGAAAAATTATCACAACTGATGCGATGGGTTGCCAGAAAGATATTGCAGAGAAGATACAAAA**

**AB811624.1** **................................................................................**

**GU299792.1** **................................................................................**

**EU549863.1** **................................................................................**

12970 12980 12990 13000 13010 13020 13030 13040

....|....|....|....|....|....|....|....|....|....|....|....|....|....|....|....|

**U18-41-new** **ACGGGGGAGGTGATTATTTATTCGCTGTAAAAGTAAATTAGGGGCGGCTTAATAAAGCCATTGAGGGATAATCCCCGCTG**

**AB811624.1** **................................................................................**

**GU299792.1** **................................................................................**

**EU549863.1** **................................................................................**

13050 13060 13070 13080 13090 13100 13110 13120

....|....|....|....|....|....|....|....|....|....|....|....|....|....|....|....|

**U18-41-new** **AAAGAATTAAATAATCCAAAGCATGGCAGTTACGAAATAAGTGAACAGAGTCACGGCAGAGAAGACATCCGTCTTCATAT**

**AB811624.1** **................................................................................**

**GU299792.1** **................................................................................**

**EU549863.1** **................................................................................**

13130 13140 13150 13160 13170 13180 13190 13200

....|....|....|....|....|....|....|....|....|....|....|....|....|....|....|....|

**U18-41-new** **TGTTTGCGATGTTCCTGATAAACTTATTGATTTCACGTTTTAATGGAAAGGACTGATAAAATTATGCGTGGCAGTCTCCT**

**AB811624.1** **................................................................................**

**GU299792.1** **................................................................................**

**EU549863.1** **................................................................................**

13210 13220 13230 13240 13250 13260 13270 13280

....|....|....|....|....|....|....|....|....|....|....|....|....|....|....|....|

**U18-41-new** **TTCGGTCAATAATAGCAGAACAAAAGAAGGAGCCTGAAATGACGGTCAGATATTATATCAGTTCTGCGGGTTTAACAGCA**

**AB811624.1** **................................................................................**

**GU299792.1** **................................................................................**

**EU549863.1** **................................................................................**

13290 13300 13310 13320 13330 13340 13350 13360

....|....|....|....|....|....|....|....|....|....|....|....|....|....|....|....|

**U18-41-new** **GAGAAGTTTGCGACAGTGATTCGAAATCACTGGCACGTGAAAAATAAGCTGCACTGGCGTCAGGGCGTGGTAATGAATGA**

**AB811624.1** **................................................................................**

**GU299792.1** **................................................................................**

**EU549863.1** **................................................................................**

13370 13380 13390 13400 13410 13420 13430 13440

....|....|....|....|....|....|....|....|....|....|....|....|....|....|....|....|

**U18-41-new** **AGACCACTGCAAAATAAGAAGAGGAATTGCAGCAGAATTATTTTCAGGGATCAGGTATATCGGCATTAATATTTTGACGA**

**AB811624.1** **................................................................................**

**GU299792.1** **................................................................................**

**EU549863.1** **................................................................................**

13450 13460 13470 13480 13490 13500 13510 13520

....|....|....|....|....|....|....|....|....|....|....|....|....|....|....|....|

**U18-41-new** **AACATAAAGTATTCAAGGCTGGGTTAAGATGTAAGATGAGAAGAGCAGATATGGACAGAGACTACCTCGCATCAGTTTTT**

**AB811624.1** **................................................................................**

**GU299792.1** **................................................................................**

**EU549863.1** **................................................................................**

13530 13540 13550 13560 13570 13580 13590 13600

....|....|....|....|....|....|....|....|....|....|....|....|....|....|....|....|

**U18-41-new** **GCGGGGAGCTGGCTTTCGTAATCTTGCCCTGGTGATGCAATGCTGAATTAATAGCATTAAAGATTTTAAATAAAATGGTT**

**AB811624.1** **................................................................................**

**GU299792.1** **................................................................................**

**EU549863.1** **................................................................................**

13610 13620 13630 13640 13650 13660 13670 13680

....|....|....|....|....|....|....|....|....|....|....|....|....|....|....|....|

**U18-41-new** **TAACTCAACATGAACTTTATAATTATTATTAGAATGATTTTGCCTTTCTGTATTTTTTTGAGTATTTTACTTTACTCTGG**

**AB811624.1** **................................................................................**

**GU299792.1** **..........................................................-.....................**

**EU549863.1** **................................................................................**

13690 13700 13710 13720 13730 13740 13750 13760

....|....|....|....|....|....|....|....|....|....|....|....|....|....|....|....|

**U18-41-new** **AAGGGTGGATAGTGTTTTTTTATTTTCTATTGAATACACATTTATAAGTGTGGCAATCTTTCTGATAAAGAGAAAGTTTG**

**AB811624.1** **................................................................................**

**GU299792.1** **................................................................................**

**EU549863.1** **G...............................................................................**

13770 13780 13790 13800 13810 13820 13830 13840

....|....|....|....|....|....|....|....|....|....|....|....|....|....|....|....|

**U18-41-new** **TAAGTAATATTTTATGGGTAATTTATCTTTCCATTTTAGGTGTGCAGGCAGCGTCGCTATTTAGTTCAGGACAATATTTG**

**AB811624.1** **................................................................................**

**GU299792.1** **................................................................................**

**EU549863.1** **................................................................................**

13850 13860 13870 13880 13890 13900 13910 13920

....|....|....|....|....|....|....|....|....|....|....|....|....|....|....|....|

**U18-41-new** **ATGCCTCTTGCCTTGTCGAATGCTGCAGAAATTGAATCCTTGGGTATTGGTGAGGTAATTAAAGTTTTTTTTGTTTTTAT**

**AB811624.1** **................................................................................**

**GU299792.1** **................................................................................**

**EU549863.1** **................................................................................**

13930 13940 13950 13960 13970 13980 13990 14000

....|....|....|....|....|....|....|....|....|....|....|....|....|....|....|....|

**U18-41-new** **TTTGTTTATTATAATTTCATTCATTACCTTGCCTTCAGGGGCTCTAACCGAGCGTATTAAATATTATAAATTCAACCTTC**

**AB811624.1** **................................................................................**

**GU299792.1** **................................................................................**

**EU549863.1** **................................................................................**

14010 14020 14030 14040 14050 14060 14070 14080

....|....|....|....|....|....|....|....|....|....|....|....|....|....|....|....|

**U18-41-new** **TCTGTATAGTATTATTGCTCGTGTGTTCTTTCATAAGTGCGGGACCAGTATATGCTTTTTATGAAACAGCAAAATTGTTC**

**AB811624.1** **................................................................................**

**GU299792.1** **................................................................................**

**EU549863.1** **................................................................................**

14090 14100 14110 14120 14130 14140 14150 14160

....|....|....|....|....|....|....|....|....|....|....|....|....|....|....|....|

**U18-41-new** **TATTTACAGCAAACCTTTAAGCCTAGTAAGAAGATCGGAGATGCAGGGAAATTCTTTTTAAAAACAAGTGTTTTTATTAA**

**AB811624.1** **................................................................................**

**GU299792.1** **................................................................................**

**EU549863.1** **................................................................................**

14170 14180 14190 14200 14210 14220 14230 14240

....|....|....|....|....|....|....|....|....|....|....|....|....|....|....|....|

**U18-41-new** **CCCTATAGAAAACAACTACAGTTTCAAAGGCCGTAATGTTATTGTAATATTTGCTGAGGGATTTTCATCTGAAATTATGG**

**AB811624.1** **................................................................................**

**GU299792.1** **................................................................................**

**EU549863.1** **................................................................................**

14250 14260 14270 14280 14290 14300 14310 14320

....|....|....|....|....|....|....|....|....|....|....|....|....|....|....|....|

**U18-41-new** **GTAATGCCAAACCAAGAGCATTTTCAGTTACGCCTAATTTAGATAAATTATCTACTGAAGCACTAAGTTTCAAAAATTAT**

**AB811624.1** **................................................................................**

**GU299792.1** **................................................................................**

**EU549863.1** **................................................................................**

14330 14340 14350 14360 14370 14380 14390 14400

....|....|....|....|....|....|....|....|....|....|....|....|....|....|....|....|

**U18-41-new** **TATAACCATACAGCTGCAACATTCCGAGGATTAAGAGGTCAGTTAACTTCTGGTTATCAATTTCGAGATGGTTTAACTGA**

**AB811624.1** **................................................................................**

**GU299792.1** **................................................................................**

**EU549863.1** **................................................................................**

14410 14420 14430 14440 14450 14460 14470 14480

....|....|....|....|....|....|....|....|....|....|....|....|....|....|....|....|

**U18-41-new** **CGCTGGCACAGGTATCGCACAATTATCTAAAGCAGAAATTAATAATATTTACCTTCACCGTCAAACATCTTTACCTGATA**

**AB811624.1** **................................................................................**

**GU299792.1** **................................................................................**

**EU549863.1** **................................................................................**

14490 14500 14510 14520 14530 14540 14550 14560

....|....|....|....|....|....|....|....|....|....|....|....|....|....|....|....|

**U18-41-new** **TATTAAAAGAGTATGGATATAAAAGTTATTTTATAGCTTCCACCGAAAAAAATAGCCCACTCAATACAATGTTGAAGACA**

**AB811624.1** **................................................................................**

**GU299792.1** **................................................................................**

**EU549863.1** **................................................................................**

14570 14580 14590 14600 14610 14620 14630 14640

....|....|....|....|....|....|....|....|....|....|....|....|....|....|....|....|

**U18-41-new** **CTTAATTTTGATAAGGTTCTTGGTATGGGTGATTTTATTGGTTATCAACGAGACAGAATGACGGATAAGCAAACATTTAA**

**AB811624.1** **................................................................................**

**GU299792.1** **................................................................................**

**EU549863.1** **................................................................................**

14650 14660 14670 14680 14690 14700 14710 14720

....|....|....|....|....|....|....|....|....|....|....|....|....|....|....|....|

**U18-41-new** **TGCACTGCAATATTTTTTACGTTCTCAGGAAAATAAAAAAGGACCCTTCTTTATTGGTGTCTATCCATCTGGTACTCATC**

**AB811624.1** **................................................................................**

**GU299792.1** **................................................................................**

**EU549863.1** **................................................................................**

14730 14740 14750 14760 14770 14780 14790 14800

....|....|....|....|....|....|....|....|....|....|....|....|....|....|....|....|

**U18-41-new** **ATGGTCAAGATAGCCCAAATGAAAAATATTTTGATGGTAACAACCCTCTCTACAATAAGTTCTATAACTATGATTTTCAG**

**AB811624.1** **................................................................................**

**GU299792.1** **................................................................................**

**EU549863.1** **................................................................................**

14810 14820 14830 14840 14850 14860 14870 14880

....|....|....|....|....|....|....|....|....|....|....|....|....|....|....|....|

**U18-41-new** **TTAGGGAAATTTATTGACTTCTTTCGGCGCAGTTCATTTTATAATAATACATTATTAATAATAACGTCAGATCATTCAAC**

**AB811624.1** **................................................................................**

**GU299792.1** **................................................................................**

**EU549863.1** **................................................................................**

14890 14900 14910 14920 14930 14940 14950 14960

....|....|....|....|....|....|....|....|....|....|....|....|....|....|....|....|

**U18-41-new** **ATTTCCTTCGACAGAATATAAAAAAGCATTTAATAGCGATTCTCGTTACTTTGTAGGCGAAATACCATTTCTGGTTATTG**

**AB811624.1** **................................................................................**

**GU299792.1** **................................................................................**

**EU549863.1** **................................................................................**

14970 14980 14990 15000 15010 15020 15030 15040

....|....|....|....|....|....|....|....|....|....|....|....|....|....|....|....|

**U18-41-new** **GAGGAGATATTACTCCTGAAATTTTGGATGCAGACGGTAAAAATTCATTAAGCTTTGCGCCAACAATATTGCATATGTTA**

**AB811624.1** **................................................................................**

**GU299792.1** **................................................................................**

**EU549863.1** **................................................................................**

15050 15060 15070 15080 15090 15100 15110 15120

....|....|....|....|....|....|....|....|....|....|....|....|....|....|....|....|

**U18-41-new** **GGGATTCAGTATTCAATGAACTATTTTTTGGGATGTTCTCTTATTGATAAGGAATGCACATCGCCATTTTCACATCTTTC**

**AB811624.1** **..................................................................GANCTG..GGCA.G**

**GU299792.1** **................................................................................**

**EU549863.1** **................................................................................**

15130 15140 15150 15160 15170 15180 15190 15200

....|....|....|....|....|....|....|....|....|....|....|....|....|....|....|....|

**U18-41-new** **TGCTATTGGTAGTGACTATTTCGTTACAAGCAAAGAAAATCATGTTGAACTAATACCAAACAATAATGATAGTGAATTAA**

**AB811624.1** **CAA-----------------------------------------------------------------------------**

**GU299792.1** **................................................................................**

**EU549863.1** **................................................................................**

15210 15220 15230 15240 15250 15260 15270 15280

....|....|....|....|....|....|....|....|....|....|....|....|....|....|....|....|

**U18-41-new** **TAAAGCTTGCAGAAAGTTTCTATAATATCAGTGGATAAGCAGATGGTTGGGCAAAAAATAATCGCCTCATCTAATTATGA**

**AB811624.1** **--------------------------------------------------------------------------------**

**GU299792.1** **................................................................................**

**EU549863.1** **................................................................................**

15290 15300 15310 15320 15330 15340 15350 15360

....|....|....|....|....|....|....|....|....|....|....|....|....|....|....|....|

**U18-41-new** **AACATACGACTGTAAAATAAGATATAAATTAAGCTATTCTTTATCTTAAATAAGCCGCAACCCCGCGGTGACCACCCCTG**

**AB811624.1** **--------------------------------------------------------------------------------**

**GU299792.1** **.-..............................................................................**

**EU549863.1** **................................................................................**

15370 15380 15390

....|....|....|....|....|....|....|...

**U18-41-new** **ACAGGAGTAAACAATGTCAAAGCAACAGATCGGCGT--**

**AB811624.1** **--------------------------------------**

**GU299792.1** **..................C.................AG**

**EU549863.1** **....................................CG**
